# A statin-regulated microRNA represses human c-Myc expression and function

**DOI:** 10.1002/emmm.201101045

**Published:** 2012-08-07

**Authors:** Apana A L Takwi, Yan Li, Lindsey E Becker Buscaglia, Jingwen Zhang, Saibyasachi Choudhury, Ae Kyung Park, Mofang Liu, Ken H Young, Woong-Yang Park, Robert C G Martin, Yong Li

**Affiliations:** 1 Department of Biochemistry and Molecular Biology, School of Medicine, University of LouisvilleLouisville, KY, USA; 2 Division of Surgical Oncology, Department of Surgery, School of Medicine, University of LouisvilleLouisville, KY, USA; 3 Department of Medicine, School of Medicine, University of LouisvilleLouisville, KY, USA; 4 Department of Pharmacy, Sunchon National University College of PharmacySunchon, Korea; 5 State Key Laboratory of Molecular Biology, Institute of Biochemistry and Cell Biology, Shanghai Institutes for Biological Sciences, Chinese Academy of SciencesShanghai, China; 6 Department of Hematopathology, The University of Texas MD Anderson Cancer CenterHouston, TX, USA; 7 Department of Biomedical Sciences, Seoul National University, College of MedicineSeoul, Korea

**Keywords:** c-Myc, lovastatin, medulloblastoma, microRNA, miR-33b

## Abstract

c-Myc dysregulation is one of the most common abnormalities found in human cancer. MicroRNAs (miRNAs) are functionally intertwined with the c-Myc network as multiple miRNAs are regulated by c-Myc, while others directly suppress c-Myc expression. In this work, we identified miR-33b as a primate-specific negative regulator of c-Myc. The human *miR-33b* gene is located at 17p11.2, a genomic locus frequently lost in medulloblastomas, of which a subset displays c-Myc overproduction. Through a small-scale screening with drugs approved by the US Food and Drug Administration (FDA), we found that lovastatin upregulated miR-33b expression, reduced cell proliferation and impaired c-Myc expression and function in miR-33b-positive medulloblastoma cells. In addition, a low dose of lovastatin treatment at a level comparable to approved human oral use reduced tumour growth in mice orthotopically xenografted with cells carrying *miR-33b*, but not with cells lacking *miR-33b*. This work presents a highly promising therapeutic option, using drug repurposing and a miRNA as a biomarker, against cancers that overexpress c-Myc.

## INTRODUCTION

The *MYC* gene encodes the c-Myc transcription factor that binds the consensus hexanucleotide sequence 5′-CAC[C/T]GTG, termed enhancer boxes (E-boxes), to activate or repress a great number of genes (Amati et al, 2001; Eilers & Eisenman, 2008; Grandori et al, 2000). These genes encompass a broad range of biological and pathological functions (Patel et al, 2004), such as cell cycle progression, apoptosis, proliferation (Bouchard et al, 2004; Gatti et al, 2009; Podar et al, 2006; Stearns et al, 2006; Teleman et al, 2008), migration and metastasis (Ma et al, 2010), stem cell self-renewal (Smith et al, 2010) and enhanced somatic reprogramming (Nakagawa et al, 2008). Dysregulation of c-Myc through somatic mutation, chromosomal translocation, genomic amplification or defects in upstream regulators (Albihn et al, 2010) plays a significant role in human cancer development (Couillard & Trudel, 2009; Jensen et al, 2003; Shi et al, 2009). MicroRNAs (miRNAs) are a group of 20–25 nucleotide small RNA molecules that repress gene expression through interaction with the 3′ untranslated region (3′UTR) of target mRNAs. Over 1000 miRNAs have been found in the human genome (Griffiths-Jones et al, 2006), rendering miRNAs one of the largest classes of regulatory molecules. Over a dozen miRNAs such as the miR-17–92 cluster (He et al, 2005; O'Donnell et al, 2005) and miR-9 (Ma et al, 2010) have been found to be induced by c-Myc to manifest its function in cell cycle, survival, metabolism, apoptosis and metastasis (Bui & Mendell, 2010). Moreover, miR-145 (Sachdeva et al, 2009), miR-34a (Christoffersen et al, 2010), miR-24 (Lal et al, 2009), miR-141 (Zhang et al, 2010), miR-185-3p (Liao & Lu, 2011) and let-7 (Melton et al, 2010) are found to repress c-Myc expression directly and adversely affect c-Myc's oncogenic function. In embryonic stem cells, c-Myc regulates miR-141, miR-200a and miR-429 to attenuate stem cell differentiation (Lin et al, 2009). These studies strongly support that miRNAs are integral components of the c-Myc network, and modulating their expression represents a potent novel approach for cancer therapeutics.

Most, if not all, miRNA translational researchers entertain the idea of using modified DNA and/or RNA oligonucleotides as the main weapons to restrain the action of oncogenic miRNAs or to mimic tumour-suppressive miRNAs. However, in addition to the common challenges of drug development, there are three intrinsic disadvantages of this approach. First, these oligonucleotides are too large in size. Experimental modified oligonucleotides tested in rodents and primates are generally longer than 15 bases (MW ∼5000 Da) (Elmen et al, 2008; Krutzfeldt et al, 2005). In contrast, the MW of most orally active drugs for human use is no greater than 500 Da, as summarized by Lipinski's Rule of Five (Lipinski et al, 2001), a rule of thumb to evaluate druglikeness. Second, DNA or RNA oligonucleotides are likely to be regarded by the human body as a virus, thereby triggering an immune response. For example, Fomivirsen (Vitravene®), the first and the only modified oligonucleotide approved by the US Food and Drug Administration (FDA) to treat cytomegalovirus retinitis in immunocompromised patients, has a well-known side effect of ocular inflammation (The Vitravene Study Group, 2002). Third, modified antisense oligonucleotides are themselves likely to act as miRNAs that could target hundreds, if not thousands, of transcripts in humans, as only seven or eight nucleotides are needed for miRNAs to repress their targets efficiently (Bartel, 2009).

In this study, we pursue novel applications of FDA-approved drugs to modulate the expression of a c-Myc-targeting miRNA. We first found that miR-33b is a negative regulator of c-Myc through direct binding to the 3′UTR of the *MYC* mRNA. miR-33b overexpression leads to down-regulation of c-Myc and its transactivational targets. The *miR-33b* allele is located at 17p; loss of this locus is the most commonly reported cytogenetic change in medulloblastoma (de Bont et al, 2008; Seranski et al, 1999), a tumour type with c-Myc overproduction (de Bont et al, 2008). Re-expression of miR-33b in a cell line without endogenous *miR-33b* decreases c-Myc protein levels, reduces anchorage-independent growth, and attenuates orthotopic xenografts in immuno-deficient mice. Through a small-scale screening with FDA-approved compounds, we found that lovastatin, a small natural chemical (MW 404 Da), increases the expression of miR-33b and down-regulates c-Myc expression and function in medulloblastoma cells with an endogenous *miR-33b* gene; lovastatin treatment also attenuates the growth of tumours orthotopically xenografted with these cells. As lovastatin is a renowned well-tolerated medicine to lower cholesterol with staggering safety records, this study supports a promising regimen against c-Myc-additive tumours using today's medicine rather than oligonucleotide-based miRNA replacement therapies that require lengthy pre-clinical and clinical testing.

## RESULTS

### Identification of miR-33b as a negative regulator of c-Myc

**Figure 1:**
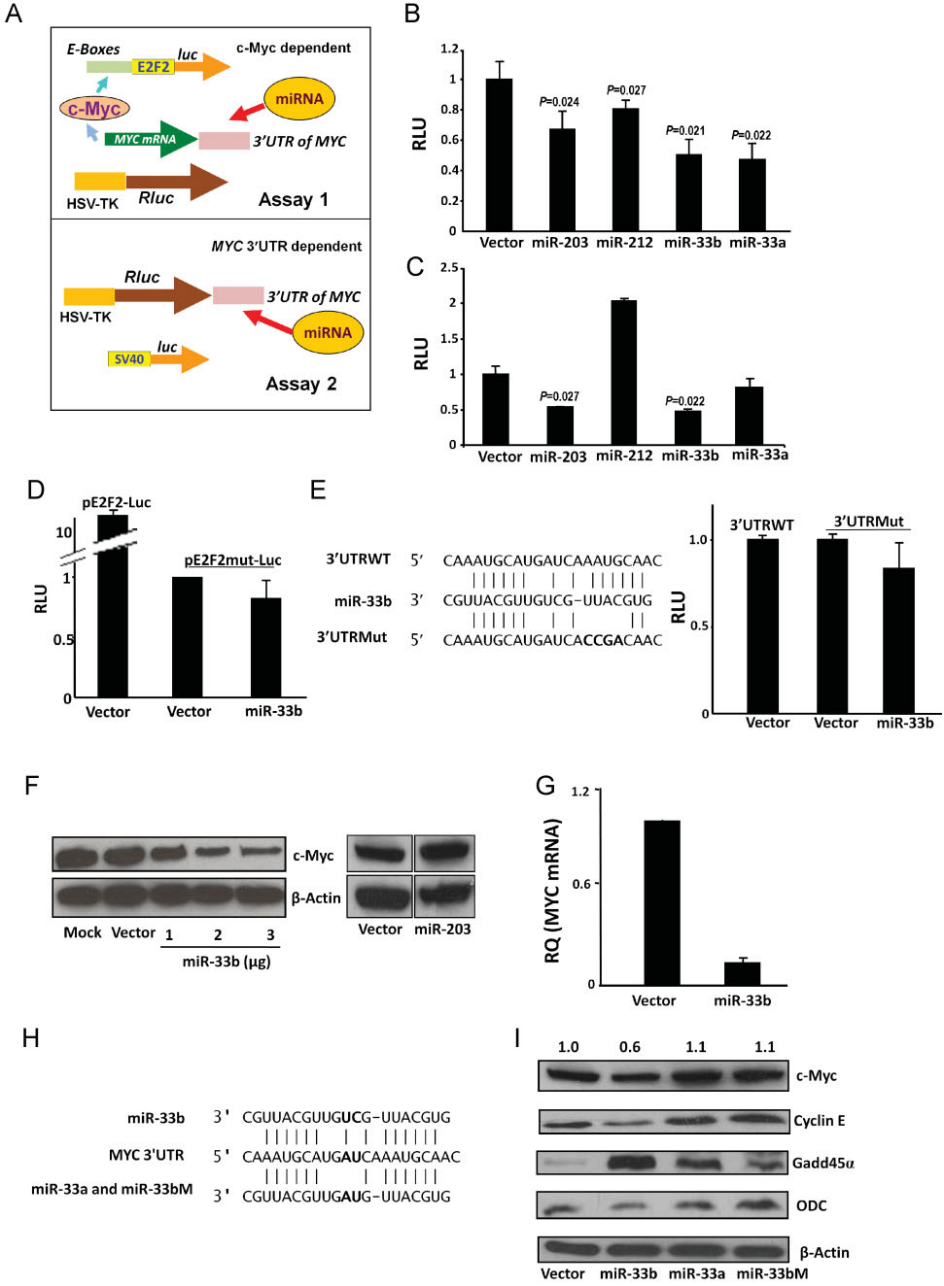
miR-33b negatively regulates c-Myc expression through direct targeting of its 3′UTR in 293T cells Reporter screening to identify miRNAs that repress c-Myc and target the 3′UTR of the *MYC* gene.Assay 1 identifies miRNAs that down-regulate the expression of *luc*, which is driven by the *E2F2* promoter. The *y*-axis denotes the relative luminescence units (RLU) of *luc* normalized to that of *Rluc* from pRL-TK compared with that of the vector control.miR-33b down-regulates the expression of *Rluc* upstream of the 3′UTR of *MYC* driven by a constitutively active promoter in Assay 2. The *y*-axis denotes the RLU of *Rluc* normalized to that of *luc* from the pGL3-Promoter compared with that of the vector control.Mutating two E-boxes of the *E2F2* promoter abolishes the regulation of *luc* expression by miR-33b in Assay 1. RLU of cells with both the parental vector and the mutant promoter construct (pE2F2Mut-luc) is used as a reference.Modulation of *Rluc* expression by miR-33b is abolished with a mutant *MYC* 3′UTR. On the left is a schematic representation of miR-33b complementary binding to *MYC* 3′UTRWT and 3′UTRMut in which the miR-33b binding site is compromised. Assay 2 (right) is performed with RLU of cells transfected with the parental vector plus 3′UTRWT or that with the parental vector plus 3′UTRMut as a reference.Immunoblotting analyses show that down-regulation of c-Myc by miR-33b is dose dependent and miR-203 does not reduce c-Myc protein levels.qRT-PCR shows that *MYC* mRNA levels are reduced when miR-33b is overexpressed.Schematic representation of the binding of miR-33b, a miR-33b mutant (miR-33bM), or miR-33a to the *MYC* 3′UTR.c-Myc and its transcriptional targets, cyclin E and ODC, are down-regulated by miR-33b, but not miR-33a or miR-33bM, while GADD45α is up-regulated. Numbers across the top of the blot indicate the relative levels of c-Myc normalized to β-actin.

We used reporter assays to screen miRNAs for their ability to modulate c-Myc function and to target the *MYC* 3′UTR directly (Fig 1A). In Assay 1, a firefly luciferase gene (luc) is driven by the E2F transcription factor 2 promoter (E2F2-luc), which is regulated by c-Myc (Sears et al, 1997). miRNAs of interest, E2F2-luc, and a Renilla luciferase (*Rluc*) construct constitutively expressing *Rluc* were co-introduced into 293T cells to screen miRNAs that down-regulate the reporter *luc*. In Assay 2, we cloned the 3′UTR of *MYC* downstream of the *Rluc* construct and co-expressed it with a respective miRNA and a constitutively expressed *luc* to determine whether the miRNA targets the *MYC* 3′UTR to down-regulate *Rluc*. We performed Assay 1 in 293T cells using hundreds of miRNA minigenes in our genetic library (Lu et al, 2011) and found that 4 miRNAs (miR-33a, miR-33b, miR-212 and miR-203) significantly down-regulated the c-Myc-dependent reporter (Fig 1B; Supporting Information Fig S1A). Next, we performed Assay 2 using 54 miRNAs that were predicted to target *MYC* and found that miR-33b and miR-203 down-regulated the reporter with the *MYC* 3′UTR downstream (Fig 1C; Supporting Information Fig S1B). To determine whether *MYC* is a *bona fide* miR-33b target gene, we performed Assay 1 using a mutant E2F2-luc construct in which two of its four E-boxes (c-Myc binding sites) were disrupted (Sears et al, 1997). As expected, with the perturbation of these two E-boxes, the expression of *luc* was significantly reduced, and the regulation of *luc* by miR-33b was abolished (Fig 1D). When a *MYC* 3′UTR mutation that disrupts its binding to the seed sequence of miR-33b was used in Assay 2, we found that the down-regulation of *Rluc* by miR-33b was abrogated (Fig 1E). Overexpression of miR-33b down-regulated c-Myc protein levels in a dose-dependent manner (Fig 1F). We did not pursue miR-203, as it did not reduce c-Myc protein levels. We noted that two reported MYC-targeting miRNAs—miR-34a (Christoffersen et al, 2010) and let-7 (Melton et al, 2010) scored negatively in our assays (Supporting Information Fig S1), suggesting the limitation of this single cell line screening. The steady-state levels of *MYC* mRNA were significantly reduced with miR-33b overexpression (Fig 1G), indicating that mRNA degradation likely contributed to miR-33b-mediated *MYC* suppression. It is noteworthy that c-Myc expression was down-regulated by miR-33b, but not miR-33a, which differs from miR-33b by only two nucleotides (UA compared with CG) in the middle of their mature sequences (Fig 1H). We folded the precursors of miR-33b and miR-33a (Zuker, 2003) and found the structure of pre-miR-33b was more stable than that of pre-miR-33a (Supporting Information Fig S2A). When the miR-33b and miR-33a minigenes were introduced into 293T cells, miR-33b was overexpressed ∼25-fold, while miR-33a was overexpressed approximately fivefold. It is likely the less stable pre-miR-33a leads to lower levels of mature miR-33a, which is supported by the finding that disruption of the stem of pre-miRNAs significantly reduces the efficiency of miRNA maturation (Han et al, 2006). We constructed a mutant miR-33b (miR-33bM) minigene with a mature sequence the same as miR-33a and a precursor similar to pre-miR-33a. When equal amounts of miR-33b and miR-33bM minigenes were introduced into 293T cells, miR-33bM was overexpressed approximately fivefold, instead of ∼25-fold. Correspondingly, introduction of miR-33b, but not miR-33a or miR-33bM, reduced the protein levels of c-Myc, as well as two known c-Myc transactivational targets, cyclin E and ornithine decarboxylase (ODC). On the other hand, Gadd45α, a c-Myc-repressed target, was upregulated when miR-33b was overexpressed (Fig 1I). Collectively, these data implicate that miR-33b is a *bona fide* negative regulator of c-Myc in our assays.

The miR-33b binding site is present in *MYC* 3′UTRs from human, chimpanzee, and rhesus, but not those from mouse, rat, dog, and other mammals; the *miR-33b* gene is only present in primates (Supporting Information Fig S2B) (Griffiths-Jones et al, 2006), suggesting that miR-33b is a primate-specific regulator of c-Myc. Overexpression of an exogenous *MYC* gene in 293T cells did not increase the expression levels of miR-33b (Supporting Information Fig S3), indicating that miR-33b expression is unlikely to be regulated by c-Myc. To further verify that miR-33b specifically targets *MYC,* we introduced two c-Myc constructs into a *MYC*-null cell line, HO15.19 (Mateyak et al, 1997); both constructs have a native c-Myc coding sequence, but one has a wild-type 3′UTR (3′UTRWT) and the other has a mutant 3′UTR in which the miR-33b binding site was disrupted (3′UTRMut, Fig 1D). As shown in Supporting Information Fig S4A, miR-33b down-regulated the expression of c-Myc and cyclin E in HO15.19 cells with 3′UTRWT, but not in those with 3′UTRMut. The changes of *MYC* mRNA levels showed similar patterns to that of the protein levels (Supporting Information Fig S4B). In addition, miR-33b led to increased G1 arrest in HO15.19 cells carrying *MYC* with 3′UTRWT (Supporting Information Fig S4C).

### miR-33b regulates c-Myc expression and function in medulloblastoma cells

**Figure 2:**
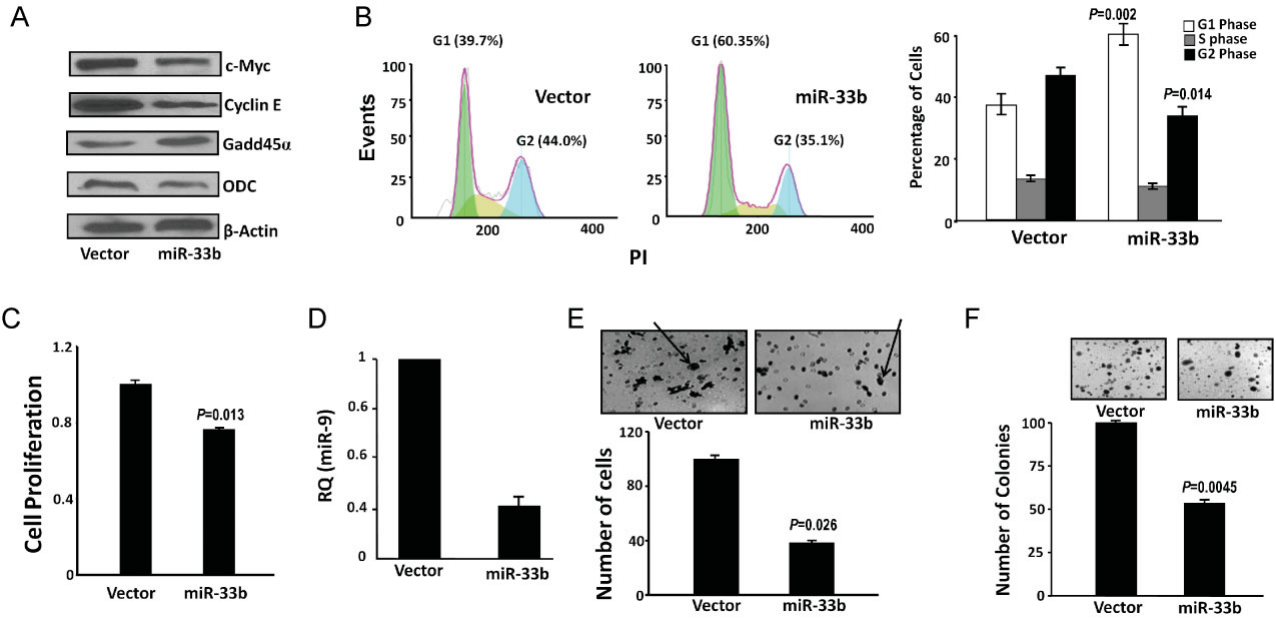
Reintroduction of miR-33b in D283 medulloblastoma cells down-regulates c-Myc expression and function Protein levels of c-Myc and its transcriptional targets cyclin E and ODC are down-regulated by miR-33b. GADD45α, a c-Myc transrepressional target, is upregulated.miR-33b leads to increased G1 arrest. On the left is a representative image of a single run with the *y*-axis denoting events (the number of cells) and the *x*-axis denoting the emitted fluorescence of the DNA dye (PI); a bar graph on the right is provided to summarize the three independent runs.miR-33b decreases cell proliferation. miR-33b expression reduces cell proliferation in the presence of exogenously expressed c-Myc with a native but not with a mutant 3′UTR.miR-33b overexpression results in down-regulation of miR-9.miR-33b reduces cell migration. The upper panel is a representative image of migrated cells; the bottom panel is a bar graph summarizing three independent experiments.miR-33b decreases anchorage-independent colony formation.

The human *miR-33b* gene is located in intron 17 of the *sterol regulatory element binding transcription factor 1* (*SREBF1*) gene at the genomic locus 17p11.2, which is frequently lost in medulloblastoma (Aldosari et al, 2000; Frühwald et al, 2001; Seranski et al, 1999). There are two transcript isoforms of this gene (*SREBF1a* and *SREBF1c*) and both contain miR-33b. We first employed D283 Med (D283), a medulloblastoma cell line without 17p11.2 and with high levels of c-Myc, but without *MYC* gene amplification (Siu et al, 2003). When miR-33b was reintroduced, c-Myc expression was down-regulated, along with its transactivation targets cyclin E and ODC, while Gadd45α (a c-Myc-repressed target) was upregulated (Fig 2A). Along with down-regulation of cyclin E, a greater percentage of D283 cells were arrested at the G1 phase when miR-33b was overexpressed compared with the vector control (Fig 2B). In addition, cells proliferated at a slower rate with miR-33b overexpression as determined by the MTT assay (Fig 2C). miR-9 is a transactivational target of c-Myc and regulates cell migration and tumour metastasis (Ma et al, 2010). We found that miR-33b reintroduction down-regulated the expression of miR-9 (Fig 2D) and resulted in a reduction in cell migration (Fig 2E). In addition, D283 cells with miR-33b overexpression formed significantly fewer colonies on soft agar (Fig 2F). These results suggest that reintroduction of miR-33b represses c-Myc expression and function in D283 cells.

**Figure 3:**
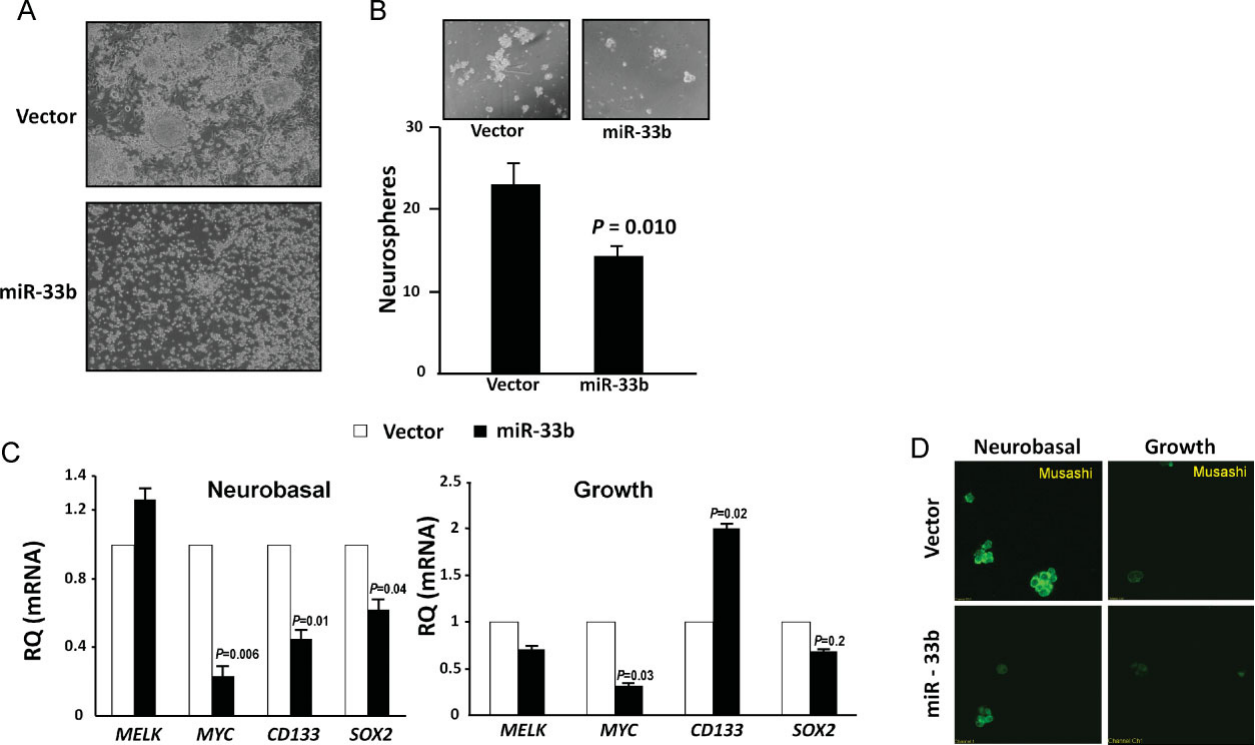
Reintroduction of miR-33b in D283 cells reduces the neuronal stem-cell characteristics Morphological change of D283 cells with stable expression of miR-33b in growth medium.Neurosphere formation of D283 cells with miR-33b stimulation is impaired in neurobasal medium.Differential mRNA expression levels of stem cell markers CD133 and SOX2 in cells with or without miR-33b in neurobasal medium (left) or growth medium (right).Immunocytometry shows that miR-33b expression results in reduced expression of Musashi in neurobasal medium.

Medulloblastomas have been reported to have a stem cell origin and are capable of forming neurospheres when incubated in neurobasal medium (Annabi et al, 2008; Hemmati et al, 2003). Even in regular growth medium, there is a major morphological change (multicell aggregates become desegregated single-cell suspensions) in D283 cells stably expressing miR-33b (Fig 3A). When cultured in neurobasal medium, D283 cells expressing miR-33b formed fewer neurospheres compared to the control (Fig 3B). In neurobasal medium, the mRNA levels of *MYC* and two stem cell markers, *SRY* (sex determining region Y)-box 2 (*SOX2*) and *CD133* (i.e., Prominin 1), were reduced with miR-33b overexpression, while in growth medium, miR-33b introduction resulted in decreased expression of *MYC,* but not of CD133 and *SOX2* (Fig 3C). The expression of maternal embryonic leucine zipper kinase (MELK), a key regulator of neural stem cell proliferation (Nakano et al, 2005), was upregulated with miR-33b overexpression. We also determined the expression of another neural stem cell marker Musashi, an RNA-binding protein that is essential for neurosphere formation and proliferation (Kaneko et al, 2000; Sakakibara et al, 1996, 2002). miR-33b-expressing D283 neurospheres had lower levels of Musashi. When cells were cultured in growth medium, Musashi expression, albeit low, was not altered significantly (Fig 3D). These data support that miR-33b has a negative impact on the morphology and neurosphere formation of D283 cells.

### Lovastatin upregulates miR-33b expression in medulloblastoma cells

**Figure 4:**
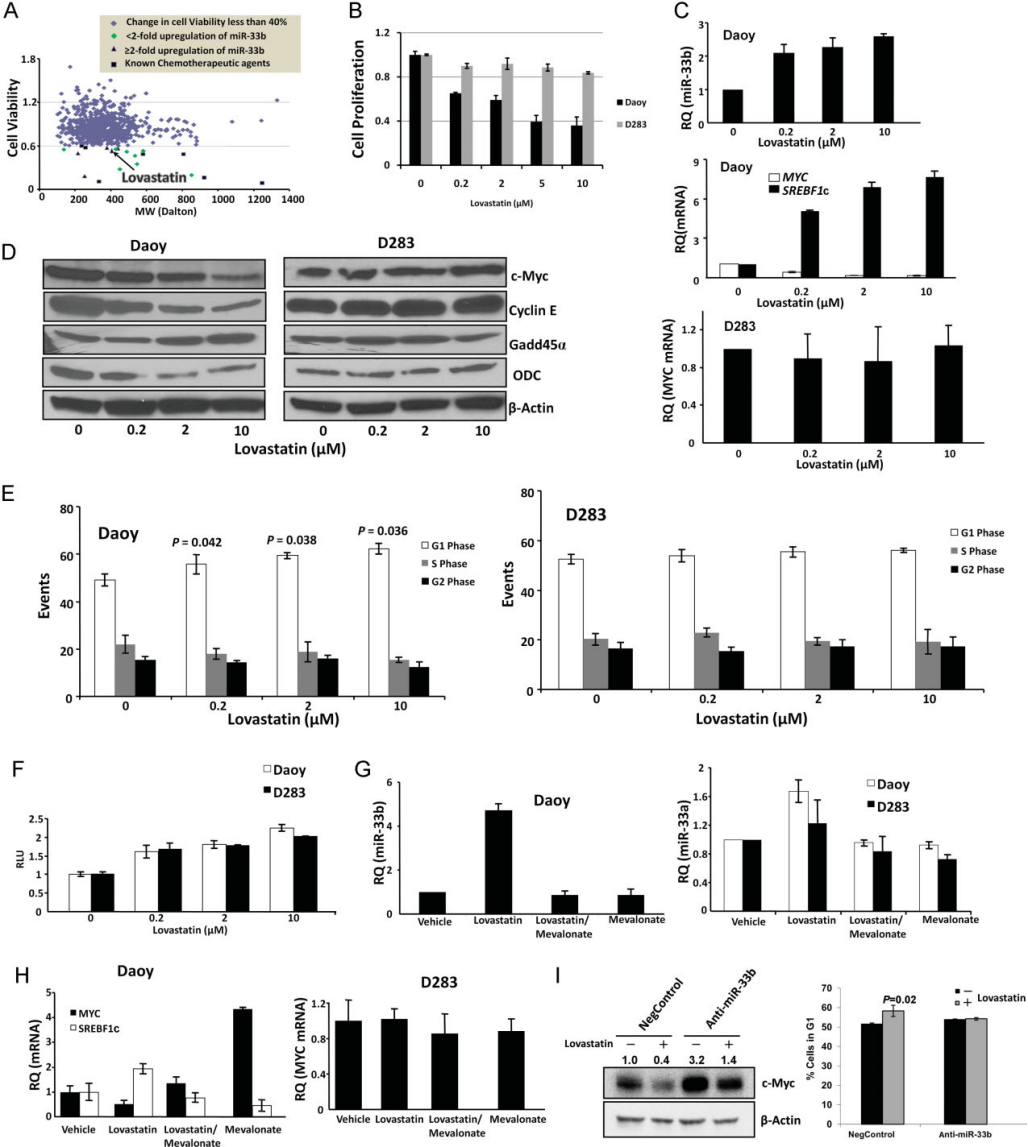
Lovastatin upregulates miR-33b expression and adversely impacts c-Myc expression and function in medulloblastoma cells A screening assay identified lovastatin as a compound that inhibits Daoy cell growth.Lovastatin treatment inhibits the growth of Daoy but not D283 cells.Lovastatin treatment increases the RNA levels of miR-33b and *SREBF1* and reduces that of *MYC* in Daoy but not in D283 cells.Lovastatin results in down-regulation of c-Myc, cyclin E, and ODC and upregulation of Gadd45α in Daoy but not in D283 cells.Cell cycle analyses of Daoy (left) and D283 (right) cells treated with lovastatin, with the final concentration of lovastatin (0–10 µM) indicated on the *x*-axis.Lovastatin treatment activates the luciferase reporter driven by the *SREBF1∼miR-33b* promoter in Daoy and D283 cells.Mevalonate inhibits lovastatin-induced miR-33b and miR-33a expression.Mevalonate inhibits c-Myc down-regulation and SREBF1 upregulation induced by lovastatin in Daoy cells.miR-33b inhibition rescues c-Myc down-regulation by lovastatin treatment. NegControl, Negative Control #1; Anti-miR-33b, Anti-miR™ miR-33b inhibitors (Ambion). Left: c-Myc expression using Western blot (numbers indicating the relative c-Myc levels normalized to β-actin); Right: G1 cell cycle arrest analysis using flow cytometry.

We performed a small-scale screening assay to identify FDA-approved compounds that reduce medulloblastoma cell viability and increase miR-33b expression using Daoy, a medulloblastoma cell line with an intact 17p11.2 and no gene amplification of *MYC* (Stearns et al, 2006). Out of 727 chemicals, 12 reduced the viability of Daoy cells ≥40% and increased miR-33b expression ≥2-fold (Fig 4A and Supporting Information Table S1). Among the five compounds that have not been indicated for cancer therapeutics, we singled out lovastatin due to the following reasons. (i) Its safety and efficacy have been tested for over 30 years. (ii) Statins are reported to reduce cancer risk, though the absolute risk reduction is likely low and there is evidence against their roles in cancer prevention (Demierre et al, 2005; Poynter et al, 2005). (iii) Statins or other cholesterol-lowering approaches upregulate miR-33a, the homolog of miR-33b (Horie et al, 2010; Marquart et al, 2010; Najafi-Shoushtari et al, 2010; Rayner et al, 2010). (iv) Treating brain tumours requires the penetration of the blood–brain barrier (BBB), a separation of circulating blood and cerebrospinal fluid in the central nervous system; lovastatin is lipophilic and is able to penetrate the BBB, which comes with some side effects such as sleep disturbances (Botti et al, 1991; Guillot et al, 1993; Maron et al, 2000). Lovastatin did not affect the proliferation of D283 cells (Fig 4B). A qPCR assay showed that in Daoy cells, lovastatin induced miR-33b and *SREBF1* (1c and 1a) expression in a dose-dependent manner, while *MYC* mRNA was down-regulated (Fig 4C, top and middle; Supporting Information Fig S5A). As expected, there was no significant change in *MYC* mRNA levels upon lovastatin treatment in D283 cells (Fig 4C, bottom). Correspondingly, lovastatin caused reduction in c-Myc and cyclin E protein levels and upregulation of Gadd45α in Daoy but not in D283 cells (Fig 4D). Cell cycle analyses demonstrated that a larger percentage of Daoy but not D283 cells were arrested at the G1 phase upon lovastatin treatment (Fig 4E). We also treated UW228 cells, another medulloblastoma cell line without 17p11.2 abnormality or *MYC* amplification (Stearns et al, 2006), with lovastatin and found there was a significant induction of miR-33b and *SREBF1c* and a decrease in c-Myc expression (Supporting Information Fig S6). Other statins also upregulated miR-33b and inhibited Daoy growth, but less significantly (Supporting Information Fig S5B). We tested the promoter of the human *SREBF1∼miR-33b* gene with a reporter assay and found that lovastatin increased its activity in both Daoy and D283 cells (Fig 4F). In addition, mevalonate inhibited the induction of miR-33b/a and *SREBF1c* and c-Myc down-regulation by lovastatin in Daoy cells. Lovastatin also slightly induced miR-33a expression but did not change *MYC* mRNA levels and mevalonate inhibited miR-33a induction in D283 cells (Fig 4G and H). These data suggest that lovastatin activates miR-33b through the cholesterol biosynthetic pathway.

Like lovastatin treatment, exogenous miR-33b overexpression in Daoy cells resulted in down-regulation of c-Myc, cyclin E and ODC and upregulation of Gadd45α (Supporting Information Fig S7A), as well as decreased *MYC* mRNA levels (Supporting Information Fig S7B). In addition, miR-33b overexpression led to a larger percentage of Daoy cells arrested at the G1 phase (Supporting Information Fig S7C), decreased cell proliferation (Supporting Information Fig S7D), and lowered miR-9 expression (Supporting Information Fig S7E) along with reduced cell migration (Supporting Information Fig S7F). We noted that the overexpression of miR-33b ranged from ∼2- to 10-fold when determined 6–48 h posttransfection, higher than that with lovastatin treatment (approximately 2-fold, Fig 4C). We also subjected Daoy cells with or without miR-33b overexpression to the neurosphere formation assay and found that no neurospheres were formed. Nonetheless, these results demonstrate that miR-33b overexpression and lovastatin treatment have a similar impact on c-Myc expression and function in Daoy cells.

To determine whether the negative effect of miR-33b on cell proliferation is due to c-Myc repression, we attempted to restore c-Myc expression in medulloblastoma cells using exogenous c-Myc constructs with or without c-Myc 3'UTR. Yet c-Myc protein levels remained unchanged in all three tested medulloblastoma cell lines: D283, Daoy and UW288, despite of successful c-Myc overexpression in HO15.19 cells (Supporting Information Fig S4; c-Myc was undetectable in naive HO15.19 cells) and many other cell lines using the same plasmid or its derivatives (Ricci et al, 2004). We next turned to HeLa cells, in which miR-33b down-regulated c-Myc expression, reduced the levels of cyclin E and ODC, and upregulated Gadd45α (Supporting Information Figs S8A and S8B). miR-33b overexpression increased G1 cell cycle arrest (Supporting Information Fig S8C) and inhibited cell proliferation in HeLa cells (Supporting Information Fig S8D). Importantly, the negative impact of miR-33b on cell proliferation of this cell line was reversed by the introduction of an exogenous *MYC* gene with a 3'UTR that cannot be targeted by miR-33b, but not by that with a WT 3'UTR (Supporting Information Fig S8D). This suggests that miR-33b-triggered reduction of cell proliferation is mediated by c-Myc down-regulation in HeLa cells.

To establish the causative effect of c-Myc down-regulation mediated by miR-33b upon lovastatin treatment, we introduced antisense miR-33b inhibitors (Anti-miR-33b) into Daoy cells prior to lovastatin treatment. As shown in Fig 4I, the expression of c-Myc was upregulated with miR-33b inhibition without lovastatin treatment. More importantly, c-Myc down-regulation by lovastatin was rescued when miR-33b inhibitors were added (comparing lane 4–2 in the left panel, Fig 4I). In addition, lovastatin-induced G1 cell cycle arrest was also abolished with miR-33b inhibition (right panel, Fig 4I). These data support the causative relationship between miR-33b overexpression and c-Myc repression upon lovastatin treatment in Daoy cells.

It should be noted that there are genes other than *MYC* negatively regulated by miR-33 and that lovastatin, like any other drug, impacts the expression of numerous genes beyond inhibiting HMG-CoA reductase. Abca1 and Pim1 are two reported miR-33 target genes (Horie et al, 2010; Ibrahim et al, 2011; Marquart et al, 2010; Najafi-Shoushtari et al, 2010; Rayner et al, 2010; Thomas et al, 2012). We found that miR-33b overexpression down-regulated the protein levels of Abca1 and Pim1 in both Daoy and D283 cells (Supporting Information Figs S9A and S9C), demonstrating the efficacy of target repression. Potential therapeutic benefits of statin therapy in medulloblastoma were reported to be associated with Bcl2 and apoptosis (Bar et al, 2007; Macaulay et al, 1999; Wang & Macaulay, 1999, 2003). We found that Bcl2 was upregulated by lovastatin treatment in Daoy, but not in D283 cells (Supporting Information Figs S9B and S9D); when miR-33b was overexpressed, Bcl2 levels were reduced in Daoy cells, but were increased in D283 cells (Supporting Information Figs S9A and S9C). In addition, the expression of Abca1 was down-regulated with lovastatin treatment in Daoy cells but not in D283 cells, while that of Pim1 was down-regulated in D283 cells but not in Daoy cells (Supporting Information Figs S9B and S9D). These data suggest the expression of these genes is impacted by unknown confounders other than miR-33b upon lovastatin treatment. We monitored cellular apoptosis using flow cytometry when Daoy or D283 cells were treated with lovastatin (10 µM) or transfected with miR-33b and found little apoptosis in either cell lines. When lovastatin concentration was increased to 40 µM, elevated apoptosis was observed in both cell lines with Daoy having a stronger response, which is in agreement with a previous report that Daoy is more sensitive to lovastatin than D283 (Dimitroulakos et al, 2001).

### A low dose of lovastatin reduces xenograft medulloblastoma growth

**Figure 5:**
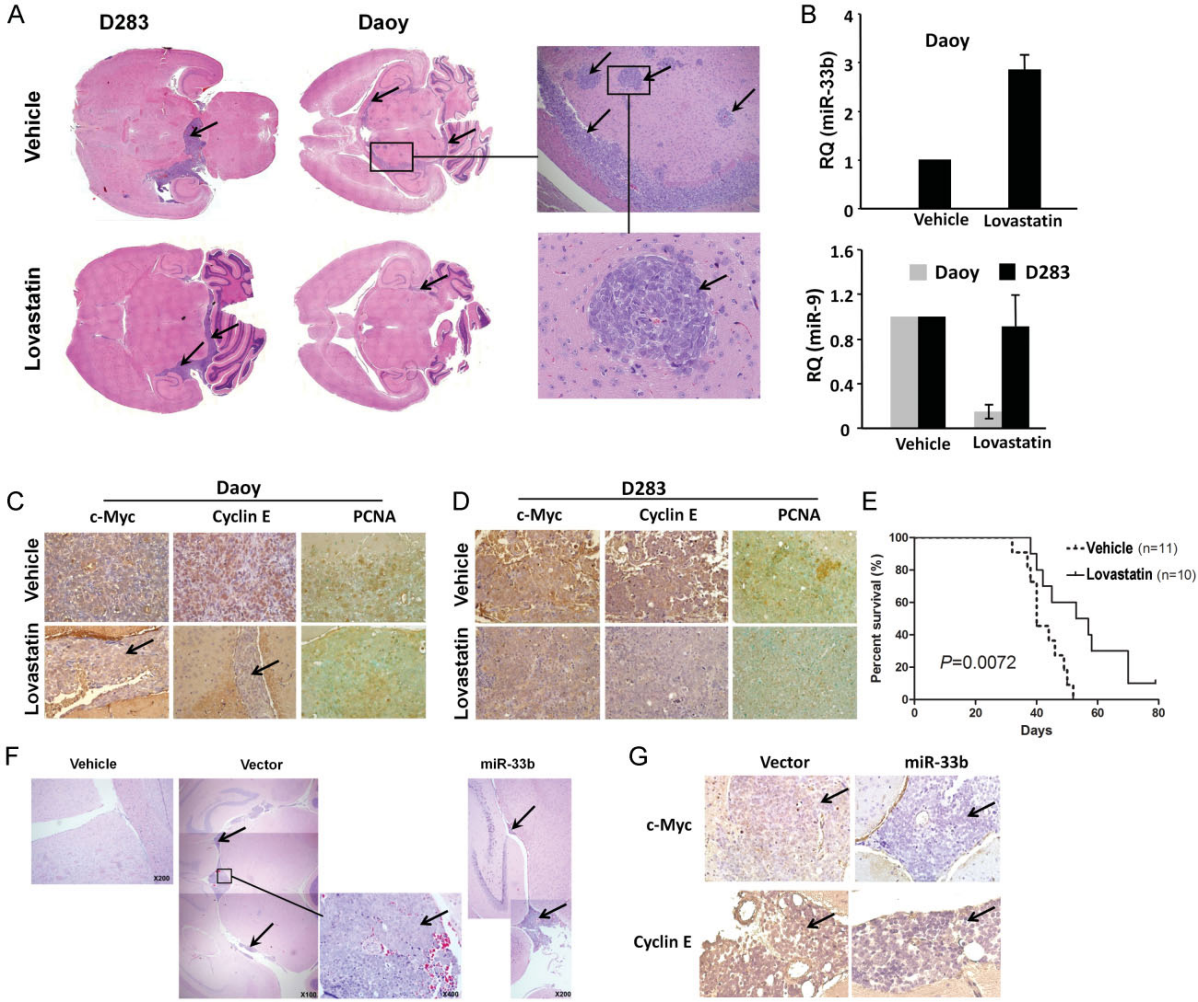
Xenograft models of medulloblastoma with lovastatin treatment or miR-33b overexpression **A.** Haematoxylin and eosin (H&E) staining of the brain from mice orthotopically xenografted with Daoy or D283 cells and treated with lovastatin.**B.** miR-33b expression was elevated and miR-9 was down-regulated in tumours with Daoy cells upon lovastatin treatment.**C,D.** IHC analyses of tumours with Daoy (**C**) or D283 (**D**) cells.**E.** Kaplan-Meier survival of mice xenografted with Daoy cells and treated with lovastatin. Treatment was administered 2 weeks after inoculation.**F.** H&E staining of tumours xenografted with D283 cells carrying miR-33b or the parental vector.**G.** IHC analyses of tumours with D283 cells carrying miR-33b or the parental vector. Arrows point to tumour cells.

We first treated mice subcutaneously xenografted with Daoy cells and found that either 1.0 or 0.2 mg/kg of lovastatin resulted in smaller tumours and lower c-Myc expression (Supporting Information Fig S10). We then tested whether lovastatin reduces the brain tumour burden in an orthotopic model. We inoculated Daoy or D283 cells into the brain of immunodeficient mice and treated them with 1.0 mg/kg lovastatin three times per week for 4 weeks. At the end of the regimen, all Daoy-bearing mice in the control group became moribund or paralyzed, yet mice in the lovastatin group appeared physically normal. We sacrificed all animal and found that Daoy-xenografted mice treated with lovastatin had less tumour expansion in the ventricles, and, strikingly, tumour invasion into the surrounding cerebellar tissues was completely blocked (Fig 5A). In contrast, D283 xenografts neither invaded surrounding tissues nor responded to lovastatin treatment (Fig 5A; Supporting Information Fig S11A). Lovastatin treatment led to miR-33b upregulation and lowered expression of miR-9, c-Myc and cyclin E in tumours of Daoy cells, but not in tumours of D283 cells (Fig 5B–D; Supporting Information Fig S11B). There were few apoptotic cells in brain tissue sections from all four groups of mice as determined by the terminal deoxynucleotidyl transferase dUTP nick end labelling (TUNEL) assay. There was no statistically significant change in the percentage of proliferating cell nuclear antigen (PCNA)-positive cells in control tumours *versus* treated Daoy tumours (Supporting Information Fig S11C), suggesting that cell cycle arrest rather than reduced cell proliferation is the likely cause of attenuated tumour growth *in vivo*.

We also performed another set of xenograft using Daoy cells and followed mice for an extended period with lovastatin treatment (1.0 mg/kg three times per week until they died). We found that mice treated with lovastatin had significantly better survival than the control group (median survival 55 *vs*. 44 days, *p* = 0.0072; Fig 5E). Necropsy revealed that there was massive tumour expansion in the ventricle of both groups. In the control group, tumour cells amassed in both the cerebral cortex (Supporting Information Figs S12A, S12B and S12C) and cerebellum (Supporting Information Fig S12D). The widely distributed tumour nodules occupied the space of neurons, causing them to denature and swell (Supporting Information Fig S12B), and many tumour nodules had established small blood vessels (Supporting Information Fig S12C). In the cerebellum, the tumour mass occupied most of the cerebellum and destructed normal cerebellum structure (Supporting Information Fig S12D). These morphological changes likely caused the loss of mobility and ability to eat and drink in these animals before they died. In mice with lovastatin treatment, the inoculated tumour cells were less involved in the cerebral cortex (Supporting Information Fig S12E) and tumour expansion into the cerebellum was not observed.

Finally, we determined whether exogenous miR-33b expression reduces the tumorigenicity of D283 cells. There was significantly less tumour expansion in brain ventricles of mice injected with cells carrying miR-33b (Fig 5F; Supporting Information Fig S11D), and these tumours expressed a lower level of c-Myc and cyclin E compared to the control (Fig 5G; Supporting Information Fig S11E). These results indicate that the *miR-33b* gene is critical to medulloblastoma's response to lovastatin treatment.

### Negative correlation of *MYC* and *SREBF1* expression in medulloblastoma

Based on histopathology, medulloblastoma has been traditionally classified into four subtypes: classical, nodular/desmoplastic, extensive nodular and large cell-anaplastic. Recently, gene-expression profiling and gene copy number analysis have identified four major subtypes of medulloblastoma: WNT, Sonic hedgehog (SHH), Group C and Group D. Group C exhibits c-Myc overexpression or amplification, lacks WNT pathway activation, and has the worst prognosis (so called Myc-driven subtype) (Eberhart, 2012). We analyzed *SREBF1* (*SREBF1a* and *SREBF1c* were not distinguishable) and *MYC* expression in medulloblastoma from four published reports with openly available data on genetic and gene expression profiles, pathway signatures and clinical pathological features (Park et al, 2012). We did not find a negative correlation between *SREBF1* and *MYC* expression when all cases were included. WNT group is a distinct disease that arises in the dorsal brain stem and not in the cerebellum (Gibson et al, 2010). When the WNT subtype and the cases with 8q aberration were excluded (only one dataset had information on 8q aberration) (Kool et al, 2008), we found that the relative mRNA levels of *SREBF1* and *MYC* were negatively correlated in a subset of cases with high *MYC* levels (*n* = 29 in blue oval; Spearman correlation coefficient = −0.38, *p* = 0.042; Supporting Information Fig S13A). We noted that in 21 cases with survival data, patients with high *MYC* expression had poor survival. This was not statistically significant, but showed a trend (*p* = 0.073 when *SREBF1* expression is divided at the 33rd percentile; Supporting Information Fig S13B). It is noteworthy that six out of seven patients with *SREBF1* expression below the 33rd percentile died within 4 years. *SREBF1* expression was significantly lower in medulloblastoma tissues compared with that in normal brain (*p* = 2.2E-16; Student's *t*-test; Supporting Information Fig S13A). In the absence of miR-33b expression data, these results implicate the association between down-regulation of *SREBF1* (the host gene of *miR-33b*) and *MYC* overexpression and poor prognosis in medulloblastoma.

## DISCUSSION

c-Myc is a transcription factor that has been implicated in the regulation of up to one-third of human genes (Amati et al, 2001; Frank et al, 2001), placing it at the centre of many biological processes, including cell cycle, differentiation and proliferation (Bouchard et al, 2004; Gatti et al, 2009; Podar et al, 2006; Stearns et al, 2006; Teleman et al, 2008). Many human cancers express high levels of c-Myc or one of its two paralogs, n-Myc or l-Myc. c-Myc is overexpressed in 31–64% of medulloblastomas, the most common cerebella tumours of the central nervous system in children (Aldosari et al, 2002; Batra et al, 1994, 1995; Bigner et al, 1990; Eberhart et al, 2004; Herms et al, 2000). Recent gene expression profiling further isolates a group of medulloblastoma with c-Myc overexpression or amplification and this subtype of the disease has the worst survival (Eberhart, 2012). *MYC* gene amplification is found only in 5–8% of medulloblastoma (Aldosari et al, 2002; Herms et al, 2000; Stearns et al, 2006), suggesting that other mechanisms exist to account for the increased *c-Myc* expression. In this work, we have identified that miR-33b that is frequently lost in medulloblastoma negatively regulates c-Myc expression and adversely affects cell proliferation, cell cycle progression, cell migration and anchorage-independent colony formation. In addition, reintroduction of miR-33b into the medulloblastoma cell line D283 lacking the endogenous *miR-33b* gene reduces orthotopic xenograft tumour growth in immuno-deficient mice. These results suggest that *miR-33b* loss is a novel mechanism of c-Myc dysregulation in a subset of medulloblastomas and that miR-33b is a potent tumour suppressor that represses the oncogenic action of c-Myc. As medulloblastoma is thought to originate from abnormal stem cells (Blazek et al, 2007), we show that miR-33b introduction into D283 cells prompts cell morphology change and reduces neurosphere formation, accompanied by lowered expression of c-Myc and certain neural stem cell markers.

Beyond medulloblastoma (Frühwald et al, 2001; Pan et al, 2005), the loss of chromosome locus 17p11.2 has also been observed in Smith–Magenis syndrome (SMS), a complex syndrome involving intellectual disabilities, sleep disturbance, behavioural problems and other anomalies. SMS usually results from a genetic change that occurs during the formation of reproductive cells (eggs or sperm) or in early foetal development. Tumour predisposition in SMS is not well studied, yet neuroblastoma, another type of child brain tumour, was reported in an SMS patient (Hienonen et al, 2005). The RAI1 gene in 17p11.2 is responsible for most features of SMS (Girirajan et al, 2006), yet the contribution of miR-33b to variable features and overall severity of the SMS syndrome remains elusive.

Despite c-Myc's role as a major oncogene and an attractive target for cancer therapeutic development for decades, attempts to identify specific chemicals to inhibit c-Myc directly have yet to produce a successful chemotherapeutic agent. Recent demonstration that JQ1, a riazolodiazepine compound specifically inhibits the bromodomain and extraterminal (BET) subfamily of human bromodomain proteins that are positive regulators of c-Myc and its downstream targets rekindles the hope of blocking c-Myc through indirect routes (Delmore et al, 2011). In the present study, we use a screening assay to identify FDA-approved chemicals that modulate the expression of miR-33b to down-regulate the expression and oncogenic activities of c-Myc. We have found that lovastatin, a natural compound first identified in the 1970s and one of the most widely used statins to lower cholesterol, upregulates the expression of miR-33b and reduces c-Myc expression and function in medulloblastoma cells (Daoy) with *miR-33b* alleles. Our results are in line with a previous report showing that statins activate the host gene *SREBF1* in some human cells (Risé et al, 2007). The effect of lovastatin on increased miR-33b RNA levels is at least partially due to activation of the genetic promoter of the *SREBF1∼miR-33b* gene. Like any other miRNAs (Baek et al, 2008), miR-33b represses the expression of multiple targets like Abca1 and Pim1, in addition to c-Myc. A recent article reports that overexpression of miR-33a or miR-33b induces a significant G1 arrest in cancer cell lines through targeting the cyclin-dependent kinase 6 (CDK6) and cyclin D1 (CCND1) genes (Cirera-Salinas et al, 2012), supporting that the miR-33 family is a regulator of cell cycle progression both directly (targeting CDK6 and CCND1) and indirectly (targeting c-Myc to reduce cyclin E expression). When miR-33b expression is inhibited in Daoy cells treated with lovastatin, c-Myc reduction is rescued and increased cell cycle arrest is reversed. This supports that miR-33b induction plays a causal role in c-Myc down-regulation by lovastatin.

Statins have an established record of human safety and efficacy in cardiovascular disease prevention and show promise for cancer prevention, yet there has been intense debate over statins' effect on cancer risk due to the limitation of observational studies and controversial results from meta-analyses (Dale et al, 2006; Demierre et al, 2005; Poynter et al, 2005). Previous studies showed that statins are effective in reducing xenograft tumour growth in mice when administered with a dose of >10 mg/kg (Laezza et al, 2008; Lin et al, 2008), which unfortunately would exceed the toxicity limit for all statins in humans (the maximum recommended therapeutic dose for lovastatin is 1.33 mg/kg/day). In our studies, lovastatin was used to treat medulloblastoma xenograft tumours with a dose of 1.0 or 0.2 mg/kg, well within the range of pharmacological stimulation for human use; this suggests that medulloblastoma may be more sensitive to lovastatin than other types of cancer. We are cognizant that lovastatin has pleiotropic effects as it may interact with diverse signalling pathways and targets, in addition to inducing miR-33b expression. At minimum, the expression of both miR-33a and *SREBF1* is upregulated by lovastatin (Fig 4): miR-33a may enhance the function of miR-33b since they share target genes (Horie et al, 2010; Marquart et al, 2010; Najafi-Shoushtari et al, 2010; Rayner et al, 2010) and the *SREBF1* gene is implied to be pro-apoptotic (Gibot et al, 2009). Our miR-33a construct did not inhibit c-Myc expression, most likely due to insufficient precursor processing (Supporting Information Fig S2A). A new article reports that miR-33a is upregulated by metformin to inhibit c-Myc expression in breast cancer cells and mouse xenografts; interestingly, this is mediated by elevated levels of DICER (Blandino et al, 2012). Therefore, activation of the transcription or enhanced processing of miR-33 to constrain the oncogenic activities of c-Myc represents one of the potential anti-cancer properties of statins or metformin. What we have documented here is the first evidence of stimulatory pharmaceutical modulation of c-Myc via a miRNA using an existing medicine with sterling safety records, which supports a new approach to miRNA-based therapeutics by way of drug repurposing.

## MATERIALS AND METHODS

### Cell culture, transfection and transduction

All cell lines were cultured at 37°C in an atmosphere containing 5% CO_2_. HEK293T, D283 and Daoy cells were obtained from ATCC (Manassas, VA) and cultured according to ATCC guidelines. c-Myc-null rat fibroblast Rat1 cells (clone HO15.19) were obtained from Dr. Claycomb's Lab (Brown University). UW228 cells were obtained from Dr. J.R. Silber (University of Washington, Seattle, WA) and were maintained in Dulbecco's modified Eagle's medium/F12 medium (Invitrogen). For neurosphere formation, D283 cells (50,000 in 6-well plates) were cultured in neurobasal medium supplemented with 10% serum, 0.1% (v/v) antibiotic–antimycotic solution (Invitrogen; Carlsbad, CA), 2 mM l-glutamine, N_2_ supplement, B27 supplement, 20 ng/ml hrEGF, 20 ng/ml hrbFGF and 50 µg/ml BSA (Invitrogen). Cells were incubated in neurobasal medium for 10 days with medium refreshed twice a week, and the plates were imaged with a microscope (magnification 10×). Spheres larger than 50 µm in diameter were counted and quantified.

Transfection was performed using Lipofectamine LTX (Invitrogen) to achieve transient expression. For miRNAs, we used the parental vector pSIF as a vector control. The dual luciferase assays were performed as described (Lu et al, 2011). For an inhibitor of miR-33b, we used Anti-miR™ miRNA inhibitor (Ambion Inc, Austin, TX) with the Negative Control #1 as a control to Anti-miR-33b. Stable expression of miRNAs was obtained using transduction with lentivirus carrying the miRNA gene or the parental vector (Lu et al, 2011). Stable expression with lentiviral infection (Lu et al, 2011) was used for neurosphere formation and xenograft experiments.

**The paper explained**

PROBLEM:

c-Myc overexpression is one of the most common abnormalities in human cancers, including a subset of medulloblastoma with the worst prognosis. Despite decades of endeavour to target the c-Myc pathway, no compound has been developed into an effective therapy in the clinic.

RESULTS:

Here we show that miR-33b, located in the genomic locus 17p11.2 that is frequently lost in medulloblastoma, is a negative regulator of c-Myc in medulloblastoma cells. Lovastatin is found to upregulate miR-33b and subsequently inhibit c-Myc expression and function. In addition, lovastatin treatment reduces tumour growth and extends survival in a mouse orthotopic xenograft model.

IMPACT:

This work supports drug repurposing to modulate miRNA expression as a therapeutic tool against c-Myc-driven medulloblastoma and other cancers. Furthermore, miR-33a, a homolog of miR-33b, was recently found to be upregulated by metformin to inhibit c-Myc expression in breast cancer cells and mouse xenografts. We suggest that miR-33 upregulation and subsequent c-Myc attenuation are critical to the anti-neoplasia action of these potential cancer prevention and treatment agents. Lovastatin and/or metformin should be directly tested in intervention studies to treat cancers with a signature of c-Myc overexpression.

### Molecular cloning and assays

The full-length *MYC* 3′UTR (467 bp) was PCR amplified utilizing a forward primer (5′-ATTCTAGAGGAAAAGTAAGGAAAACGATTCCT) and reverse primer (5′-TAGCGGCCGCGGCTCAATGATATATTTG) from A549 cell genomic DNA template. The PCR product was inserted into the *Not*I*/Xba*I site, upstream of the Renilla luciferase gene, of the pRL-TK vector (Promega). All miRNA constructs were obtained from an existing library (Lu et al, 2011), and all DNA constructs were confirmed by Sanger sequencing. The human c-Myc expression construct (pcDNA3-cmyc) was obtained from Addgene.org (#16011). We used TaqMan assays (Applied Biosystems Inc.; Foster City, CA) to determine the expression of mature miRNAs and mRNA (SREBF1c and MYC) with U6 RNA and β-actin mRNA as respective references (Chen et al, 2005). For SREBF1c, a pre-made Taqman assay was used (Hs01088683; Applied Biosystems). For SREBF1a and SREBF2, a SYBR Green based qPCR was performed (primers for SREBF1a: 5′-TAGTCCGAAGCCGGGTGGGCGCCGGCGCCAT and 5′-GATGTCGTTCAAAACCGCTGTGTGTCCAGTTC; primers for SREBF2: 5′-CACAATATCATTGAAAAGCGCTACCGGTCC and 5′-TTTTTCTGATTGGCCAGCTTCAGCACCATG.

The following primary antibodies were used in Western blotting analyses: c-Myc (cat. no. sc-40), cyclin E (sc-25303), VEGF-A (sc-7269), ODC (sc-21516) and Gadd45 (sc-6850) obtained from Santa Cruz Biotechnology (Santa Cruz, CA), Abca1 (ab7360) from Abcam (Cambridge, MA); Pim1 (#3247), and Bcl2 (#2870) from Cell Signaling Technology (Beverly, MA), and β-actin (AC-15) from Sigma (St. Louis, MO). Secondary antibodies were horseradish peroxidase-linked goat anti-mouse IgG antibody (Santa Cruz Biotechnology sc-2005, 1:5000) or goat anti-rabbit IgG antibody (Cell Signaling). The quantification of protein expression levels was determined by ImageJ (http://rsb.info.nih.gov/ij) with arbitrary units (AU) reflecting the signal density from the blot.

### Cellular assays

Cell proliferation was assessed using 3-(4,5-dimethylthiazol-2-yl)-2,5-diphenyltetrazolium bromide (MTT) (MTT Cell Proliferation Assay; ATCC, Manassas, VA). MTT reagent (0.5 mg/ml) was added to each well of transfected cells and incubated at 37°C for 4 h. Detergent reagent (100 µl) was added and incubated for 2 h in the dark at room temperature, and the absorbance was read at 570 nm. The values were normalized to their respective controls. Cell cycle phase and apoptosis were analyzed according to our established methods (Kumar et al, 2011; Lu et al, 2011). For the Transwell migration assay, we used Matrigel-coated Transwell chambers (Becton Dickinson). Transwell inserts with 8-µm pores were coated with Matrigel and reconstituted with fresh medium for 2 h before the experiment. Cells (2 × 10^4^ ml^−1^) were seeded into the upper chambers in 0.5 ml of serum-free DMEM or EMEM in 12-well plates, while 1 ml of EMEM supplemented with 10% foetal bovine serum was placed in the lower chamber. After 24 h, cells that migrated to the lower surface of the Matrigel-coated membrane were fixed with 70% ethanol and stained with 0.4% crystal violet for 2 h and counted. Non-migrated cells on the upper side of the filter were removed with a cotton swab, and the filter mounted on glass microscopic slides. The number of migrated cells was counted using ImageJ software. The results were expressed as the average number of invasive cells per microscopic field normalized to the total number of live cells cultured under the same conditions without migration. For drug screening, Daoy cells were incubated with 0.2 µM of 727 compounds from the NIH Clinical Collection 1 and 2 (Evotec; South San Francisco, CA) before being subjected to the MTT assay after 48 h. Lovastatin was used at a concentration of 0.2 –10 µM for cells and 0.2–1.0 mg/kg for mice, as serum levels of lovastatin can reach 0.10–3.92 µM in humans using ≥4 mg/kg (Thibault et al, 1996), and it is currently recommended for the treatment of hypercholesterolemia with a dose of up to 80 mg/day (or 1 mg/kg/day), which yields serum levels of ∼0.1 µM (Pan et al, 1990). Mevalonate was used at a concentration of 100 nM along with 0.2 µM of lovastatin for 24 h. For promoter activity and miR-33b/SREBF1 induction, 0.2 µM of lovastatin was used to treat cells for 24 h. All other molecular and cellular assays were performed 48 h posttreatment or posttransfection.

### Soft agar colony formation assay

Viable cells (2000) in 1.5 ml of culture medium with 1% glutamine, antibiotics, and 0.2% soft agar were layered onto 0.5% solidified agar in experimental culture media in six-well plates. The plates were incubated at 37°C for 2 weeks, the cells were stained with the MTT reagent, and colony foci were counted using a microscope (original magnification 4×). Experiments were carried out in triplicate.

### Mouse xenograft and immunohistochemistry (IHC) analyses

Animals were housed at 22–23°C in a 12-h light/dark cycle in a facility certified by the American Association for Accreditation of Laboratory Animal Care. Mice had free access to rodent chow and tap water. All animal experiments were conducted with a protocol approved by the University of Louisville Institutional Animal Care and Use Committee. Recipient immuno-deficient NCr nude mice were purchased from Taconic (Hudson, NY). Male ∼8-week-old mice (*n* = 5 in each group) were anesthetized with rapid-sequence inhalation of isofluorane before xenograft. For subcutaneous xenograft, 1 × 10^6^ Daoy cells (in 200 µl of 1× PBS) were inoculated (Lu et al, 2011); 2 weeks postinoculation, animals were randomized into two groups and lovastatin (carboxylate form; EMD4Biosciences; Darmstadt, Germany; 1.0 or 0.2 mg/kg in 20 µl of DMSO) was administered through intraperitoneal (i.p.) injection three times per week for 4 weeks. For orthotopic (intracerebellar) xenograft, mice were anesthetized with sodium pentobarbital (50 mg/kg), the head was shaved, a small skin incision (1 mm) was made along the midline, and a bore hole (0.7 mm) was created with a microsurgical drill. A 26-gauge syringe needle was used to deliver a total of 1 × 10^5^ cells (in 10 µl of 1× PBS) into the right cerebellar hemisphere. Mice were monitored daily for any sign of distress. For D283 cells stably expressing miR-33b without lovastatin treatment, mice were maintained for 6 weeks; two animals were injected with the vehicle. For lovastatin treatment, the drug was administered (1.0 mg/kg) 2 weeks after surgery through i.p. injection three times per week and was continued for 4 weeks (*n* = 6 each for animals sacrificed after 6 weeks). For xenografted animal survival analysis, mice were treated similarly till they died (*n* = 11 for the control group; *n* = 10 for the lovastatin statin). Brain tissues were fixed with 10% formalin for 24 h and embedded in paraffin.

IHC was performed using the avidin biotin complex (ABC) peroxidase method with rabbit and mouse IgG ABC Elite1 detection kits (Vector Laboratories, Burlingame, CA) as previously described (Lu et al, 2011). The rabbit polyclonal antibody to c-Myc (Santa Cruz Biotechnology; cat. no. sc-789; 1:100), cyclin E (Santa Cruz Biotechnology; cat. no. sc-25303; diluted to 1:100) and monoclonal antibody to PCNA (Santa Cruz Biotechnology; cat. no. sc-56; 1:100) were used. Tissue sections were also stained with hematoxylin and eosin. For the TUNEL assay, deparaffinized brain tissue sections were incubated with 20 µg/ml protease K for 15min at room temperature, then washed with PBS and incubated with TUNEL reaction mixture (Roche Applied Science) 60 min at 37°C in humidified atmosphere. Tissue sections were incubated with converter-POD (with anti-fluorescein antibody) and subsequently with the DAB substrate. At least three sections were stained for each specimen. For protein expression, stained slides were evaluated histologically by two independent, blinded observers, and the gradation was scored from 0 to 3 according to the intensity of staining (0, negative; 1, weak; 2, moderate; 3, strong).

### Statistical analyses

The experimental results were expressed as the mean ± standard deviation of at least three independent experiments. A two-tailed Student's *t*-test was performed with *p* ≤ 0.05 between the samples and their respective controls considered to be statistically significant. For animal survival with xenograft and lovastatin treatment, a log-rank (Mantel-Cox) test was performed.

## Supplementary material

Detailed facts of importance to specialist readers are published as ”Supporting Information”. Such documents are peer-reviewed, but not copy-edited or typeset. They are made available as submitted by the authors.
