# Supplementary material for: A statin-regulated microRNA represses human c-Myc expression and function
Source: EMBO Mol Med. 2012 Aug 7;4(9):896–909. doi: 10.1002/emmm.201101045 (PMC3491823; doi:10.1002/emmm.201101045)
Supplement: Supplementary file 1 [file emmm0004-0896-SD1.pdf]

EMBO
Molecular Medicine

Manuscript EMM-2011-01045

A Statin-regulated MicroRNA Represses Human c-Myc Expression and Function

Apana A. L. Takwi, Yan Li, Lindsey E. Becker Buscaglia, Jingwen Zhang, Saibyasachi Choudhury, Ae Kyung Park, Mofang Liu, Ken H. Young, Woong-Yang Park, Robert C. G. Martin, Yong Li

Corresponding author: Yong Li, University of Louisville

Review timeline:

Submission date:	02 November 2011
Editorial Decision:	05 December 2011
Revision received:	20 April 2012
Editorial Decision:	24 May 2012
Revision received:	29 May 2012
Editorial Decision:	01 June 2012
Revision received:	04 June 2012
Accepted:	06 June 2012

Transaction Report:

(Note: With the exception of the correction of typographical or spelling errors that could be a source of ambiguity, letters and reports are not edited. The original formatting of letters and referee reports may not be reflected in this compilation.)

1st Editorial Decision 05 December 2011

Thank you for submitting your manuscript for consideration to EMBO Molecular Medicine. Three referees have now seen it, whose comments are shown below.

As you will see, all three referees find your study of potential interest. However, they also raised significant issues mainly regarding correlative rather than causative mechanistic insight that are explicitly reported in the reports. Nevertheless I would like to particularly highlight few points that are important for our journal:

- Better *in vivo* data should be provided, better xenografts analysis (ref. #3, point 3), clarify SREB expression (ref. #1 point 1) and if available, miR-33b expression analysis in primary tumors (ref. #2)

- Mechanistic insight has to be strengthen by using gain and loss-of-function as suggested to establish causative effect (ref. #3 points 1 and 2 and ref. #1 point 2 and 3)

- Clarify miR-33a/b targeting of c- myc (ref. #1 point 4 and ref. #3 point 6) and apoptosis induction (ref. #2)

I would like to invite you to submit a revised version of the manuscript, fully addressing the comments of all three reviewers, within the time constraints outlined below. If you feel you may need extra time please let us know as soon as you can. Please note that it is EMBO Molecular Medicine policy to allow only a single round of revision and that, as acceptance or rejection of the manuscript will depend on another round of review, your responses should be as complete as possible.

Revised manuscripts should be submitted within three months of a request for revision; they will otherwise be treated as new submissions, except under exceptional circumstances in which a short extension is obtained from the editor.

I look forward to seeing a revised form of your manuscript as soon as possible.

Yours sincerely,

Editor
EMBO Molecular Medicine

***** Reviewer's comments *****

Referee #1:

In their manuscript entitled "A statin-regulated microRNA represses human c-Myc expression and functions". Apana Takwi and collaborators identify miR-33 as a statin-induced miRNA that regulates cell cycle progression and proliferation via targeting c-myc. Even though the model is interesting the mechanistic insights in this paper are somewhat limited.
Specific coments:
1. The authors shown that statin-treated Daoy cells increase the expression of miR-33b and SREBP1 in a dose -dependent manner. SREBP1a and 1c are derived from a single gene on the human chromosome 17p11.2 through the use of alternative transcription start sites that produce alternate form of exon, designated 1a and 1a. SREBP-1a is a potent activator of all SREBP-responsive genes, including those that mediate the synthesis of cholesterol, fatty acids, and triglycerides. Which SREBP-1 isoform is measured in the manuscript?, How about the levels of SREBP-2 and miR-33a in Daoy treated cells?. It is a very important question because SREBP-1a and SREBP-2 are the predominant isoforms of SREBP in most cultured cell lines, whereas SREBP-1c and SREBP-2 predominate in the liver and most other intact tissues.
2. The authors conclude that the inhibition of cell proliferation in Daoy cells treated with statins is mediated by miR-33. To support this conclusion the authors should transfected Daoy cells with miR-33b inhibitors, treat them with statins and assess proliferation and cell cycle progression.
3. It has been shown recently the miR-33 overexpression inhibit cell proliferation and cell cycle progression (Oncogene and Cancer Research 2011). In both papers the authors demonstrated the miR-33 target PIM-1. Are the PIM-1 levels different in Daoy and D283 cells treated with statins?. How about the expression of PIM-1 in cells transfected with miR-33 (Figure 1F and Figure 2A)?. If myc is the critical target of miR-33 in regulating cell proliferation, cells transfected with miR-33 and myc cDNA without the 3'UTR should recover the proliferating rate.
4. The seed sequence usually dictates the target recognition for a miRNA. It is surprising that miR-33b was able to target myc but not miR-33a. The authors should measure the expression levels of miR-33b, miR-33a and miR-33bM in cells transfected with these constructs (Figure 1I). Additional controls including the expression of other known miR-33 targets (p53, ABCA1,...) should be measured in cell transfected with miR-33b, miR-33a and miR-33bM.
5. Why miR-33b overexpression induces the expression of miR-9?. Is the transcriptional or post-transcriptional processing of miR-9 regulated by miR-33?. How miR-9 regulates cell migration?
Overall, the work presents interesting data but my main concern relates to limited mechanism presented by the authors. New data, analyzing in detail (see above) this model would strengthen the manuscript substantially.

Referee #2:

The authors present data supporting a role for miR-33b in the negative regulation of expression and activity of c-Myc in several human cell line models, including medulloblastoma. They find that treatment with lovastatin induces miR-33b and is associated with downregulation of MYC expression and cell cycle arrest in several medulloblastoma cell lines. Cells can be rescued with mevalonate, implying appropriate drug targeting. Tumor growth in vivo is inhibited either by miR-

33b expression or lovastatin treatment. The work is sound in that the experiments utilize both molecular and pharmacologic approaches which support the hypothesis that miR-33b negatively regulates c-Myc activity.

There have been several publications in the past supporting a potential therapeutic role for statin therapy in medulloblastoma (Bar EE, et al. Hedgehog signaling promotes medulloblastoma survival via BclII. Am J Pathol. 2007 Jan;170(1):347-55. PubMed PMID: 17200206; PubMed Central PMCID: PMC1762704.
Wang W, Macaulay RJ. Cell-cycle gene expression in lovastatin-induced medulloblastoma apoptosis. Can J Neurol Sci. 2003 Nov;30(4):349-57. PubMed PMID: 14672267.
Wang W, Macaulay RJ. Mevalonate prevents lovastatin-induced apoptosis in medulloblastoma cell lines. Can J Neurol Sci. 1999 Nov;26(4):305-10. PubMed PMID: 10563217.
Macaulay RJ, Wang W, Dimitroulakos J, Becker LE, Yeger H. Lovastatin-induced apoptosis of human medulloblastoma cell lines in vitro. J Neurooncol. 1999 Mar;42(1):1-11. PubMed PMID: 10360474). These studies have proposed increased apoptosis in medulloblastoma with statin treatment, potentially due to down-regulation of Bcl2. There may also be a synergistic effect of inhibition of the hedgehog pathway and statin therapy.

Interestingly, the authors of this work do not find (or do not report) increased apoptosis in statin-treated medulloblastoma cell lines. The dominant feature in this work is cell cycle arrest, arguably due to increased miR-33b expression leading to decreased c-Myc activity. While the two findings are not mutually exclusive, it would be interesting to determine if miR-33b upregulation (either in the transfected or lovastatin treated cells) also leads to increased apoptosis, testing for altered Bcl2 and/or evidence of increased apoptosis.
This work would also be supported by some assessment of miR-33b expression in primary tumors and, ideally, a correlation with ch17 alterations/loss.

Three very minor points in the figures...Figure 1 (I) has numbers across the top of the western blot, these are presumably normalized c-Myc/Actin ratios, but it is not stated in the figure legend. Figure 3(C) lacks p-values for the various expression values and there is no mention of MELK in either the text or the figure legend. And the arrows in figure 6 are a bit misleading, the tumor cells are in aggregate and extend beyond the arrows, potentially tracing around the xenograft might be more helpful.

Referee #3:

The manuscript by Takwi et al demonstrates that c-Myc is a bona fide target of the microRNA-33b (but curiously not of the closely related miR-33a) and that in medulloblastomas cell lines this targeting leads to decreased cancer cell proliferation and migration. The authors then propose that low-dose lovastatin, which appears to increase miR-33b and decrease c-Myc levels, could be used as an MB therapeutic. The concept is certainly interesting and some basic observations are solid and well-documented. Unfortunately, the manuscript in its present form is full of gaping holes, which weaken the authors' story considerably. Either these holes need to be patched, or the conclusions of the paper need to be scaled down. Obviously, only the first scenario would justify eventual publication in EMBO Mol Med.

Major points:
1. Using data in Figures 2 and 3, the authors claim that the negative effects of miR-33b on cell proliferation and other traits of the transformed phenotype are based on Myc down-regulation. This claim is rather poorly supported by data. For one, the connection between Myc down-regulation and cell migration and formation of neurospheres is purely correlative. The contribution of Myc to miR-33b-mediated cell proliferation is supported somewhat by data in panel 2C, but it raises more questions than it answers. First, what is "proliferation"? Is it total cell numbers, or percentage of cells in the S-phase, or WST units? Second, it is unclear how levels of exogenous (wt and mut) Myc compare to those of endogenous Myc. A Western blot would be very helpful in this regard, as is the inclusion of empty vector-transduced cells.
2. In Figure 3, the authors show that lovastatin increases miR-33b and decrease c-Myc levels, but again there is no attempt to establish causation. For example, if one knocks down miR-33b levels, would lovastatin still down-regulate Myc? Furthermore, if one restores Myc expression, would

lovastatin still inhibit cell growth?
3. This correlative approach to science becomes even more problematic in Figure 6, where purported anti-tumor effects of lovastatin are depicted. First, these effects need to be better quantitated, as "before and after" pictures are not compelling and are too small to interpret anyway. Direct tumor measurement or Kaplan -Meier survival curves would be more relevant. Of equal importance is the fact that there is no direct evidence that lovastatin works through miR-33b or Myc, making the central conclusion of the paper doubly weak.

Minor points:
4. There is a paucity of primary information in the paper, especially pertaining to the microRNA screen. How did know Myc targeting miRs (e.g, let-7 or miR-34) score?
5. What level of miR-33b overexpression was achieved in transfection experiments? If it was sky-high, the results need to be interpreted with utmost caution.
6. What exactly is the sequence of miR-33bM and how is it different from 33a?
7. The choice of the cell line notwithstanding, Figure 5 doesn't contain any information not already contained in Figure 2 - they should be consolidated.
8. The epidemiological connection between statins and cancer is complicated at best, and the authors should not selectively cite papers that favor their hypothesis.

1st Revision - Authors' Response 20 April 2012

We thank all reviewers for their suggestions and comments on our manuscript. This rebuttal file lists a point-by-point reply. We believe that our manuscript has been strengthened by the inclusion of new experiments and analyses that were performed in response to reviewers' critiques. New dada and analyses include Fig. 4I, Fig. 5E, Fig. S1, Fig. S2A, Fig. S5A, Fig. S8, and Fig. S11. We also analyzed published data and found that there is down-regulation of SREBF1 (the miR-33b host gene) in medulloblastoma tissues compared with normal brains and that there is a negative correction between *SREBF1* and *MYC* mRNA levels in a subset of medulloblastoma (Fig. S12).

Referee #1:

1. "Which SREBP-1 isoform is measured in the manuscript?, How about the levels of SREBP-2 and miR-33a in Daoy treated cells? "

The SREBP-1 isoform measured in the original manuscript was SREBP-1c. Both 1a and 1c isoforms of the SREBF1 contain miR-33b. We also measured Srebp1a, Srebp2, and miR-33a levels in lovastatin-treated Daoy cells by qPCR and all showed induction (Fig. 4C and 4G and Fig. S5A).

2. "The authors conclude that the inhibition of cell proliferation in Daoy cells treated with statins is mediated by miR-33. To support this conclusion the authors should transfected Daoy cells with miR-33b inhibitors, treat them with statins and assess proliferation and cell cycle progression. "

We treated Daoy cells with miR-33b inhibitors and found that c-Myc expression and G1 arrest were rescued by miR-33b inhibition (Fig. 4I). This supports that lovastatin inhibits cell cycle progression through the miR-33b-c-Myc axis.

3. " It has been shown recently the miR-33 overexpression inhibits cell proliferation and cell cycle progression (Oncogene and Cancer Research 2011). In both papers the authors demonstrated the miR-33 target PIM-1. Are the PIM-1 levels different in Daoy and D283 cells treated with statins?. How about the expression of PIM-1 in cells transfected with miR-33 (Figure 1F and Figure 2A)?. If myc is the critical target of miR-33 in regulating cell proliferation, cells transfected with miR-33 and myc cDNA without the 3'UTR should recover the proliferating rate. "

In both D283 and Daoy, Pim1 (as well as another miR-33 target Abca1) protein levels decreased in cells transfected with miR-33b (Fig. S8). Pim1 expression was down-regulated in D283 cells but

not Daoy cells upon lovastatin treatment, suggesting that there are other confounders to Pim1 expression. When a c-Myc exogenous construct was introduced into medulloblastoma cells, c-Myc protein levels remained unchanged. We performed the experiment with HO15.19 cells (a cell line without endogenous c-Myc expression) and found c-Myc expression from a c-Myc exogenous construct with a wild-type UTR, but not that with a mutant UTR was regulated by miR-33b (Fig. S4).

4. "The seed sequence usually dictates the target recognition for a miRNA. It is surprising that miR-33b was able to target myc but not miR-33a. The authors should measure the expression levels of miR-33b, miR-33a and miR-33bM in cells transfected with these constructs (Figure 1I). " The reviewer also asked other targets of miR-33.

We thank the reviewer's suggestion. We found the expression levels were different when three minigenes were introduced into 293T cells: the miR-33b construct produced more mature miRNAs than that of miR-33a or miR-33bM. This is likely due to that the miR-33b precursor is more stable than that of miR-33a or miR-33bM (Fig. S2A).

Mouse p53 was reported to be regulated by miR-33 (*Cell Cycle*. 2010 9:3277-85). However, the human p53 gene contains no miR-33b binding sites. We did examine the expression of Abca1 and confirmed that Abca1 was down-regulated by miR-33b (Fig. S8).

5. "Why miR-33b overexpression induces the expression of miR-9?. Is the transcriptional or post-transcriptional processing of miR-9 regulated by miR-33?. How miR-9 regulates cell migration? "

miR-9 is a transactivational target of c-Myc. The mechanism of miR-9 regulating cell migration has been illuminated by Ma et al (2010).

Referee #2:

1. The reviewer asked if apoptosis and Bcl2 were involved in the effect of medulloblastoma cell response to miR-33b overexpression and lovastatin treatment.

We cited the references in which Bcl2 down-regulation and apoptosis in medulloblastoma cells were observed as the reviewer pointed out (Bar et al, 2007; Macaulay et al, 1999; Wang & Macaulay, 1999; Wang & Macaulay, 2003). We examined whether there were changes in Bcl2 expression and apoptosis in medulloblastoma cells with miR-33b overexpression or lovastatin treatment. Bcl2 was upregulated by lovastatin treatment in Daoy, but not in D283 cells (Fig. S8B and S8D); when miR-33b was overexpressed, Bcl2 levels were reduced in Daoy cells, but were increased in D283 cells (Fig. S8A and S8C). This inconsistency suggests that there are other confounders of Bcl2 regulation. We performed apoptosis assay using flow cytometry and found increased apoptosis with high lovastatin concentration (40µM). At low concentration (10µM) or with miR-33b overexpression, there is no increase of apoptosis in either cell lines. We did not pursue higher lovastatin concentrations in cell lines and animal experiments as serum levels of lovastatin or its metabolites can only reach ~4µM in humans with an overdose of 4mg/kg (Thibault et al, 1996). As expected, we observed few apoptotic cells in our animal experiments with 1.0mg/kg lovastatin treatment.

2. "This work would also be supported by some assessment of miR-33b expression in primary tumors and, ideally, a correlation with ch17 alterations/loss. "

Due to the lack of tumor specimens, we were unable to assess the correlation between miR-33b and c-Myc. However, we sought help from Drs. Woong-Yang Park and Ae Kyung Park, who expertize in medulloblastoma molecular pathology. We analyzed *SREBF1* and *MYC* expression in medulloblastoma with openly available data on genetic and gene expression profiles, pathway signatures, and clinicopathological features (Kool et al, 2008). We did not find a negative correlation between *SREBF1* and *MYC* expression when all cases were included. Medulloblastomas have four major subtypes that have distinctive developmental origins and the WNT group is a

distinct disease that arises in the dorsal brain stem and not in the cerebellum (Gibson et al, 2010). When the WNT subtype and the cases with 8q aberration are excluded (Kool et al, 2008), we found that the relative levels of *SREBF1* and *MYC* were negatively correlated in a subset of cases with high MYC levels (n=29 in blue oval; Spearman correlation coefficient = −0.38, *P*=0.042; Fig. S12). We also examined *SREBF2* expression and did not find a correlation with *MYC* expression. We noted that in 21 cases with survival data, patients with high *MYC* expression have poor survival, but this is not statistically significant (*P*=0.073 when *SREBF1* expression was divided at the 33rd percentile). It is noteworthy that all 7 patients with *SREBF1* expression below the 33rd percentile died within 4 years. SREBF1 expression was significantly lower in medulloblastoma tissues compared with that in normal brain. In the absence of miR-33b expression data, these results implicate the association between SREBF1 down-regulation and c-Myc overexpression/poor prognosis.

3. "Figure 1 (I) has numbers across the top of the western blot, these are presumably normalized c-Myc/Actin ratios, but it is not stated in the figure legend. "

We added a statement in the figure legend.

4. "Figure 3(C) lacks p-values for the various expression values and there is no mention of MELK in either the text or the figure legend. "

We performed t-test on the ΔCt values and add the P values in Figure 3C. We describe MELK in the text.

5. "And the arrows in figure 6 are a bit misleading, the tumor cells are in aggregate and extend beyond the arrows, potentially tracing around the xenograft might be more helpful. "

We added more arrows to point tumor cells.

Referee #3:

1. "First, what is "proliferation"? Is it total cell numbers, or percentage of cells in the S-phase, or WST units? "

Cell proliferation was assessed using the MTT assay (MTT Cell Proliferation Kit from ATCC).

2. "In Figure 3, the authors show that lovastatin increases miR-33b and decrease c-Myc levels, but again there is no attempt to establish causation. For example, if one knocks down miR-33b levels, would lovastatin still down-regulate Myc? Furthermore, if one restores Myc expression, would lovastatin still inhibit cell growth? " and "Second, it is unclear how levels of exogenous (wt and mut) Myc compare to those of endogenous Myc. A Western blot would be very helpful in this regard, as is the inclusion of empty vector-transduced cells. "

We introduced miR-33b inhibitor into Daoy cells before treating them with lovastatin. The expression of c-Myc was upregulated with miR-33b inhibition without lovastatin treatment. More importantly, c-Myc down-regulation by lovastatin was rescued when miR-33b inhibitors were added. In addition, lovastatin-induced G1 cell cycle arrest was also abolished with miR-33b inhibition (Fig. 4I). These data suggest the causative relationship between miR-33b overexpression and c-Myc repression upon lovastatin treatment.

We tried to restore Myc expression using an exogenous vector, but the Myc protein levels remained unchanged in Daoy cells. However, our data with HO15.19 cells (a cell line without endogenous c-Myc expression) support the interaction of c-Myc and miR-33b. As shown in Fig. S4, miR-33b down-regulated the expression of c-Myc and Cyclin E in cells with a WT 3′UTR, but not in those

with a mutant 3′UTR. miR-33b led to increased G1 arrest in HO15.19 cells carrying *MYC* with the WT 3′UTRWT compared to cells with a mutant 3′UTR.

3. "This correlative approach to science becomes even more problematic in Figure 6, where purported anti-tumor effects of lovastatin are depicted. First, these effects need to be better quantitated, as "before and after" pictures are not compelling and are too small to interpret anyway. Direct tumor measurement or Kaplan -Meier survival curves would be more relevant. "

We performed another experiment with nude mice injected with Daoy cells. We monitored long-term survival of these mice (we did only four weeks in the original experiment). As shown in Fig. 5E, mice treated with lovastatin had significantly better survival than the control group (median survival 55 vs 40 days, P=0.0072; Log-rank (Mantel-Cox) Test). We also analyzed the brain pathology (Fig. S11).

4. "There is a paucity of primary information in the paper, especially pertaining to the microRNA screen. How did know Myc targeting miRs (e.g, let-7 or miR-34) score? "

We added a supplemental figure (Fig. S1) to include all miRNAs subjected to Assay 1 and Assay 2. Let-7s and miR-34s were not positively scored, suggesting limitation of our approach with a single cell line.

5. "What level of miR-33b overexpression was achieved in transfection experiments? If it was sky-high, the results need to be interpreted with utmost caution. "

We observed the increase in Daoy cells 6-48 hrs post-transfection ranged from 2- to 10-fold. This is higher than that with lovastatin treatment.

6. "What exactly is the sequence of miR-33bM and how is it different from 33a? "

The mature sequence of miR-33bM is the same as miR-33a, while its precursor resembles that of miR-33b.

7. "The choice of the cell line notwithstanding, Figure 5 doesn't contain any information not already contained in Figure 2 - they should be consolidated. "

We have taken the Reviewer's suggestion and labeled the original Figure 5 as supplemental Figure S7.

8. "The epidemiological connection between statins and cancer is complicated at best, and the authors should not selectively cite papers that favor their hypothesis. "

We stressed the complicated nature of statins in cancer epidemiology. "Statins are reported to reduce cancer risk though the absolute risk reduction is likely low and there is evidence against their roles in cancer prevention." "We recognize that the role of lovastatin in cancer prevention is controversial (Dale et al, 2006)".

2nd Editorial Decision 24 May 2012

Thank you for the submission of your revised manuscript to EMBO Molecular Medicine. We have now received the enclosed reports from the referees that were asked to re-assess it.

As you will see while reviewer #1 is now supportive, reviewer #3 is not satisfied by the revision provided. I would therefore stronlgy encourage you to address the remaining issues as convincingly as you possibly can.

Please submit your revised manuscript as soon as you will be able to in order to satisfactorily address the last pending issues, but not longer than a month. Please let me know if you anticipate further delay. I look forward to seeing a revised form of your manuscript as soon as possible.

I look forward to reading a new revised version of your manuscript as soon as possible.

Yours sincerely,

Editor
EMBO Molecular Medicine

***** Reviewer's comments *****

Referee #1:

The authors have responded all my questions. Only a minor suggestion. The paper recently published by Cirera-Salinas D Cell Cycle. 2012 Mar 1;11(5). [Epub ahead of print] should be mentioned in the text

Referee #3 (Comments on Novelty/Model System):

I remain highly skeptical of the authors' claims. What's novel here is not convincing, and what's convincing is not novel. For example, the authors cite 5 papers showing that lovastatin upregulates closely related miR-33a, but the effort to bring Myc into this pathway falls short of expectations.

Referee #3 (Other Remarks):

Takwi et al have addressed some of the issues raised during the original review. However, the most important points of the paper remain poorly supported by data.

1. The manuscript still doesn't have any data showing that the negative effects of miR-33b on cell proliferation are due to down-regulation of Myc and could be alleviated via Myc overexpression. This was the first major point in the original review, and the answer to this criticism is conspicuously absent from the rebuttal.

2. The second major point had to do with the claim that lovastatin suppresses Myc via induction of miR-33b. In the rebuttal the authors state that "c-Myc down-regulation by lovastatin was rescued when miR-33b inhibitors were added... (Fig. 4I)." However, upon close examination of the western blot in 4I this reviewer is convinced of the opposite: that lovastatin inhibits Myc even when miR-33b is taken out of equation. They start with the higher basal level of c-Myc in lane 3, but still lose 50-60% in lane 4.

3. Another piece of data that speaks to this hypothetical connection is in panel 2D, where D283 cells, which have a deletion encompassing miR-33b, don't down-regulate Myc in response to lovastatin. While this result is consistent with the central hypothesis, it by no means proves it, since we are dealing with the n=1.

2nd Revision - Authors' Response 29 May 2012

We thank all reviewers for their suggestions and comments on the first revision. This rebuttal file lists a point-by-point reply.

Referee #1:

The authors have responded all my questions. Only a minor suggestion. The paper recently published by Cirera-Salinas D Cell Cycle. 2012 Mar 1;11(5). [Epub ahead of print] should be mentioned in the text

We thank the reviewer's suggestion and include this article in our discussion. We noted that in this paper, MYC was mentioned as a predicted miR-33 target gene but was not tested.

Referee #3:

I remain highly skeptical of the authors' claims. What's novel here is not convincing, and what's convincing is not novel. For example, the authors cite 5 papers showing that lovastatin upregulates closely related miR-33a, but the effort to bring Myc into this pathway falls short of expectations.

The novelty of this work rests on

1. miR-33b is a negative regulator of c-Myc; This miRNA gene (locus 17p11.2) is lost in a subset of medulloblastoma.
2. SREBF1, the host gene of miR-33b, is down-regulated in medulloblastoma and. SREBF1 expression is inversely correlated with c-Myc expression in a subset of medulloblastoma with high c-Myc levels (Fig. S12).
3. We use drug repurposing to screening FDA-approved compounds to induce miR-33b expression and find that lovastatin increases miR-33b levels and inhibits c-Myc expression.

It is likely that miR-33a induction by lovastatin contributes to the suppression of miR-33 target genes. Yet we did not focus on miR-33a as miR-33a unlikely contributes to the etiology of c-Myc-driven medulloblastoma. There is no reported abnormality of the SREBF2 (the host gene of miR-33a) gene locus in medulloblastoma or other cancers and SREBF2 expression was not down-regulated in medulloblastoma compared to normal brain tissues.

Referee #3 (Other Remarks):

1. The manuscript still doesn't have any data showing that the negative effects of miR-33b on cell proliferation are due to down-regulation of Myc and could be alleviated via Myc overexpression. This was the first major point in the original review, and the answer to this criticism is conspicuously absent from the rebuttal.

We tried to restore c-Myc expression using an exogenous vector, but c-Myc protein levels remained unchanged in Daoy and D283 cells with multiple attempts. However, our data with HO15.19 cells (a cell line without endogenous c-Myc expression) support the interaction of c-Myc and miR-33b. As shown in Fig. S4, miR-33b down-regulated the expression of c-Myc and cyclin E in cells with a WT 3′UTR, but not in those with a mutant 3′UTR. miR-33b led to increased G1 arrest in HO15.19 cells carrying *MYC* with a WT 3′UTR.

2. The second major point had to do with the claim that lovastatin suppresses Myc via induction of miR-33b. In the rebuttal the authors state "c-Myc down-regulation by lovastatin was rescued when miR-33b inhibitors were added... (Fig. 4I)." However, upon close examination of the western blot in 4I this reviewer is convinced of the opposite: that lovastatin inhibits Myc even when miR-33b is taken out of equation. They start with the higher basal level of c-Myc in lane 3, but still lose 50-60% in lane 4.

We provide normalized c-Myc protein levels in Fig. 4I. Comparing lane 1 vs 2, lovastatin reduces c-Myc levels. Comparing lane 1 vs 3, anti-miR-33b increases c-Myc expression (presumably through miR-33b reduction). *Comparing lane 3 vs 4 as the reviewer observed, lovastatin treatment still reduces c-Myc expression. This suggests that anti-miR-33b did not completely block miR-33b repression of c-Myc; one can also say that with anti-miR-33b in Doay cells, lovastatin still increases*

endogenous miR-33b to reduce c-Myc protein levels. Comparing lane 1, 2, and 4, the inhibition of c-Myc by lovastatin (lane 2 vs 1) was rescued by miR-33b inhibition (lane 4).

3. Another piece of data that speaks to this hypothetical connection is in panel 2D, where D283 cells, which have a deletion encompassing miR-33b, don't down-regulate Myc in response to lovastatin. While this result is consistent with the central hypothesis, it by no means proves it, since we are dealing with the n=1.

We thank the reviewer to raise this legitimate question. Yet the D283 cell line is the only medulloblastoma cell line we know that has no endogenous miR-33b gene. We felt that the readers would be interested to see if lovastatin impacts Myc expression and function in this cell line.

3rd Editorial Decision 01 June 2012

Thank you very much for the submission of your revised manuscript to EMBO Molecular Medicine and for providing the Paper Explained and the new Supplementary Information files.

I read with attention your reply to Referee #3's concerns, particularly point 1. I do understand the technical difficulties met to answer this request experimentally. However, you did not reply to this point in your first revision. I am afraid that, due to the the very central nature of this issue for the manuscript, I cannot overrule this referee. Therefore, I would kindly but strongly encourage you to address this major point experimentally in order to convincingly show that the negative effects of miR-33b on cell proliferation are due to the down-regulation of c-Myc. Nevertheless, for time sake, I would perfectly understand if at this stage you would rather prefer to seek publication elsewhere.

Revised manuscripts should be submitted within three months of a request for revision; they will otherwise be treated as new submissions, except under exceptional circumstances in which a short extension is obtained from the editor.

I look forward to seeing a revised form of your manuscript as soon as possible. In case you decide to send your article somewhere else, I would appreciate if you could let us know.

I look forward to reading a new revised version of your manuscript as soon as possible.

Yours sincerely,

Editor
EMBO Molecular Medicine

3rd Revision - Authors' Response 04 June 2012

We would like to address the Point #1 from the third reviewer.

Referee #3 (Other Remarks):

1. The manuscript still doesn't have any data showing that the negative effects of miR-33b on cell proliferation are due to down-regulation of Myc and could be alleviated via Myc overexpression.

To determine whether the negative effect of miR-33b on cell proliferation is due to c-Myc repression, we attempted to restore c-Myc expression in medulloblastoma cells using c-Myc exogenous constructs with or without c-Myc 3'UTR. Yet c-Myc protein levels remained unchanged in all three tested medulloblastoma cell lines: D283, Daoy, and UW288, despite of successful c-Myc

overexpression in HO15.19 cells (Fig. S4; c-Myc was undetectable in naïve HO15.19 cells) and many other cell lines using the same plasmid or its derivatives (Ricci et al, 2004). We next turned to HeLa cells, in which miR-33b down-regulated c-Myc expression, reduced the levels of cyclin E and ODC, and upregulated Gadd45α (Fig. S8A and S8B). miR-33b overexpression increased G1 cell cycle arrest (Fig. S8C) and inhibited cell proliferation in HeLa cells (Fig. S8D). Importantly, the negative impact of miR-33b on cell proliferation of this cell line was reversed by the introduction of an exogenous *MYC* gene with a 3'UTR that cannot be targeted by miR-33b, but not by that with a WT 3'UTR (Fig. S8D). This suggests that miR-33b-triggered reduction of cell proliferation is mediated by c-Myc down-regulation in HeLa cells.

Ricci MS, Jin Z, Dews M, Yu D, Thomas-Tikhonenko A, Dicker DT, El-Deiry WS (2004) Direct Repression of FLIP Expression by c-myc Is a Major Determinant of TRAIL Sensitivity. *Molecular and Cellular Biology* 24: 8541-8555
