# Supplementary material for: A statin-regulated microRNA represses human c-Myc expression and function
Source: EMBO Mol Med. 2012 Aug 7;4(9):896–909. doi: 10.1002/emmm.201101045 (PMC3491823; doi:10.1002/emmm.201101045)
Supplement: Supplementary file 2 [file emmm0004-0896-SD2.pdf]

Supplementary Information for

Takwi *et al*, “A Statin-regulated MicroRNA Represses Human c-Myc Expression and Function”

Table of Contents

Figure S1 **Reporter screening to identify miRNAs that repress c-Myc using Assay 1 and 2** 2
Figure S2 **The miR-33b miRNA and its precursor** 3
Figure S3 **miR-33b expression is not regulated by c-Myc** 4
Figure S4 **miR-33b down-regulates exogenously expressed c-Myc in *MYC*-null HO15.19 cells** 5
Figure S5 **Statin affects gene expression in Daoy cells** 6
Figure S6 **Lovastatin treatment for UW228 cells** 7
Figure S7 **miR-33b overexpression in Daoy cells** 8
Figure S8 **miR-33b regulates c-Myc in HeLa cells** 9
Figure S9 **miR-33b regulates the expression of Abca1 and Pim1 in medulloblastoma cells** 10
Figure S10 **Subcutaneous xenograft assay of Daoy cells treated with lovastatin** 11
Figure S11 **Pathological analyses of orthotopic xenograft tumors in mice** 12
Figure S12 **Histology of mice xenografted with Daoy and treated with lovastatin** 13
Figure S13 **Negative correlation of *MYC* and *SREBF1* expression in medulloblastoma** 14
Table S1 **FDA-approved drugs that upregulate miR-33b in Daoy cells** 15

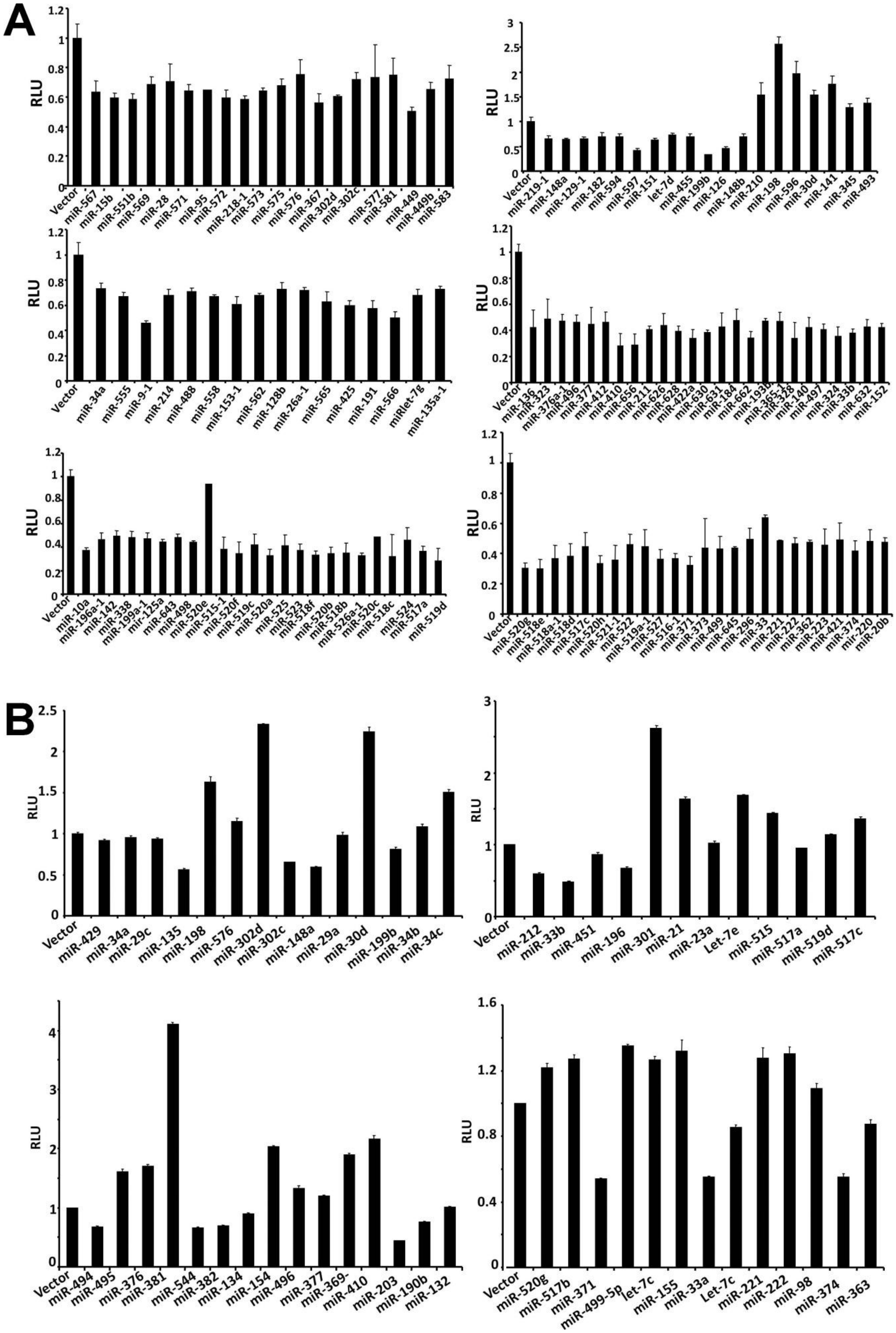

Figure S1. Reporter screening to identify miRNAs that repress c-Myc using Assay 1 (A) and 2 (B).

A

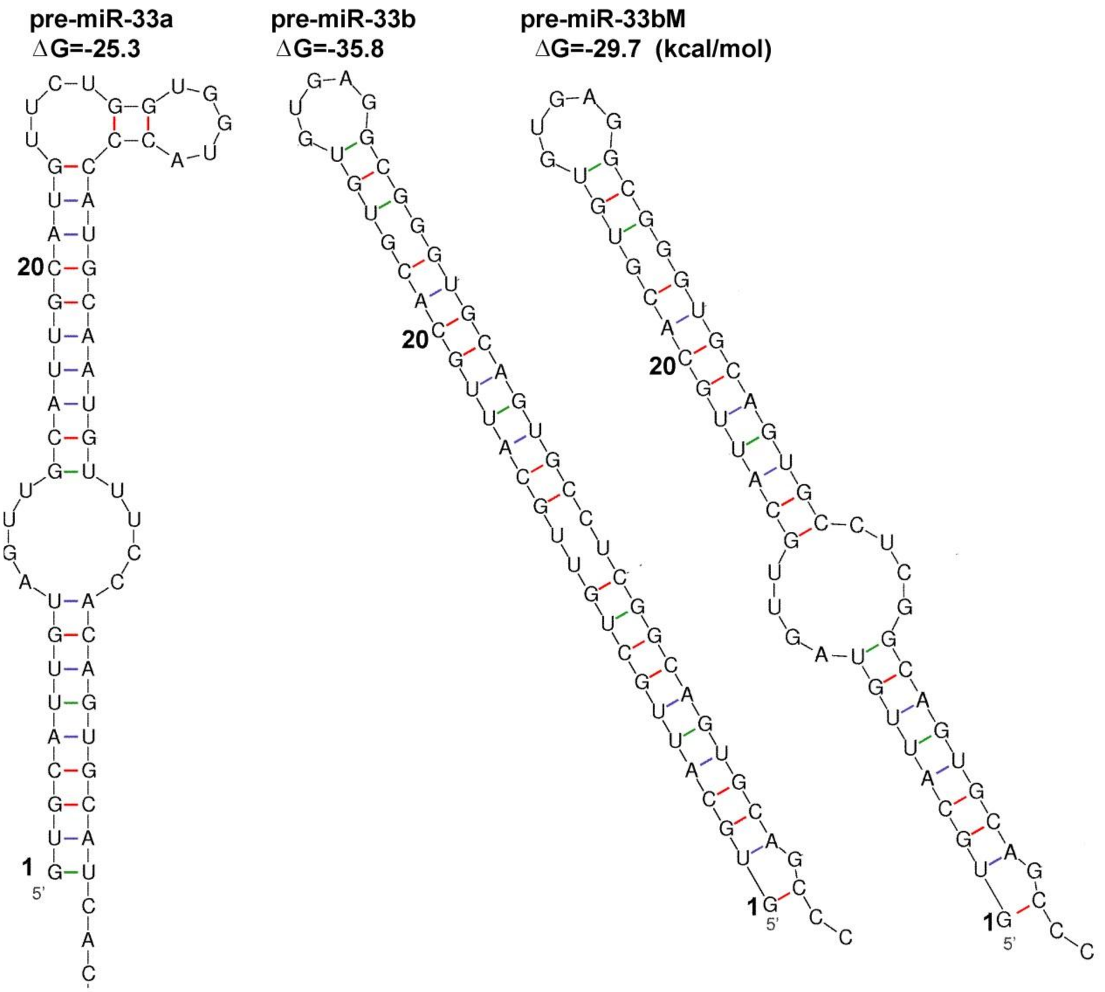

B

```
CGUUACGUUGUCG-UUACGUG  5′    hsa-miR-33b
64||||||       ||||||             101 UTR        miR-33b  miR-33a
CAAAUGCAUGAUCAAAUGCAACCUCACAACCUUGGCU   Human         +        +
CAAAUGCAUGAUCAAAUGCAACCUCACAACCUUGGCU   Chimpanzee    +        +
CAAAUGCAUGAUCAAAUGCAACCUCACAACCUUGGCU   Rhesus        +        +
-AAAUGCAUGCCAAAGCCUAACCUCACAACCUUGGCU   Mouse         –        +
-AAAUGCAUGCCAAAGCCUAACCUCACAACCUUGGCU   Rat           –        +
CACAUGCAUGGUCAAGUGCAACCUCACAACCUUGGCU   Rabbit        –        +
CAAAUGCAUGUUUAAGUGCAACCUCACAACCUUGGCU   Dog           –        +
CAAAUGCAUGGUCAAAUGUAACCUCACAACCUUGGCU   Horse         –        +
CAUAUGCAUGA------ACAACCUCACAACCUUGGCU   Cow           –        +
CAACUGCAUGCUAACUUGAGACUACACAACCUUGGCC   Chicken       –        +
CAAAUGCAUGaUcAAaUGCAACCUCACAACCUUGGCU   Consensus
```

Figure S2. The miR-33b miRNA and its precursor. (A) The predicted free energies of the precursors to miR-33b, miR-33a, and miR-33bM. Nucleotides 1-20 represent the mature form of the miRNAs. (B) The interaction of miR-33b:*MYC* 3'UTR in animals. The miR-33b gene exists in human, chimpanzee, and Rhesus, but not in other animals, even though they all have a *SREBF1, miR-33a,* and *SREBF2* gene. "+", present; "–", absent. The 64–101 nucleotide of human 3′UTR is shown.

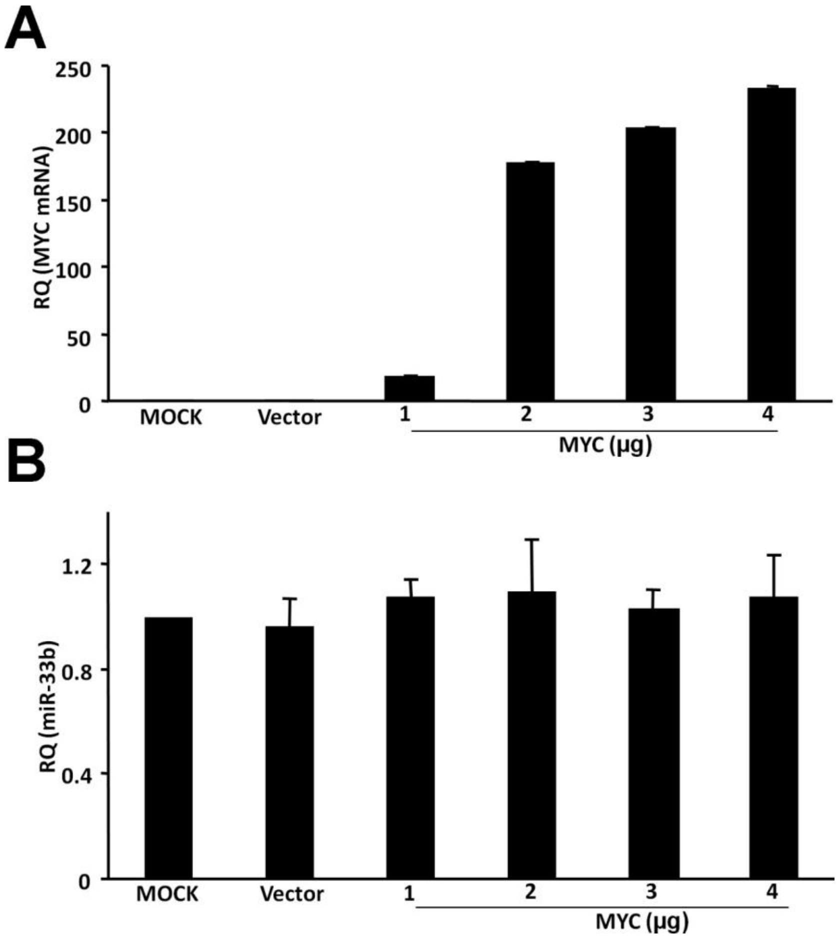

Figure S3. miR-33b expression is not regulated by c-Myc. (A) qRT-PCR demonstrates increased expression of MYC. (B) The expression of miR-33b remains unchanged upon c-Myc overexpression.

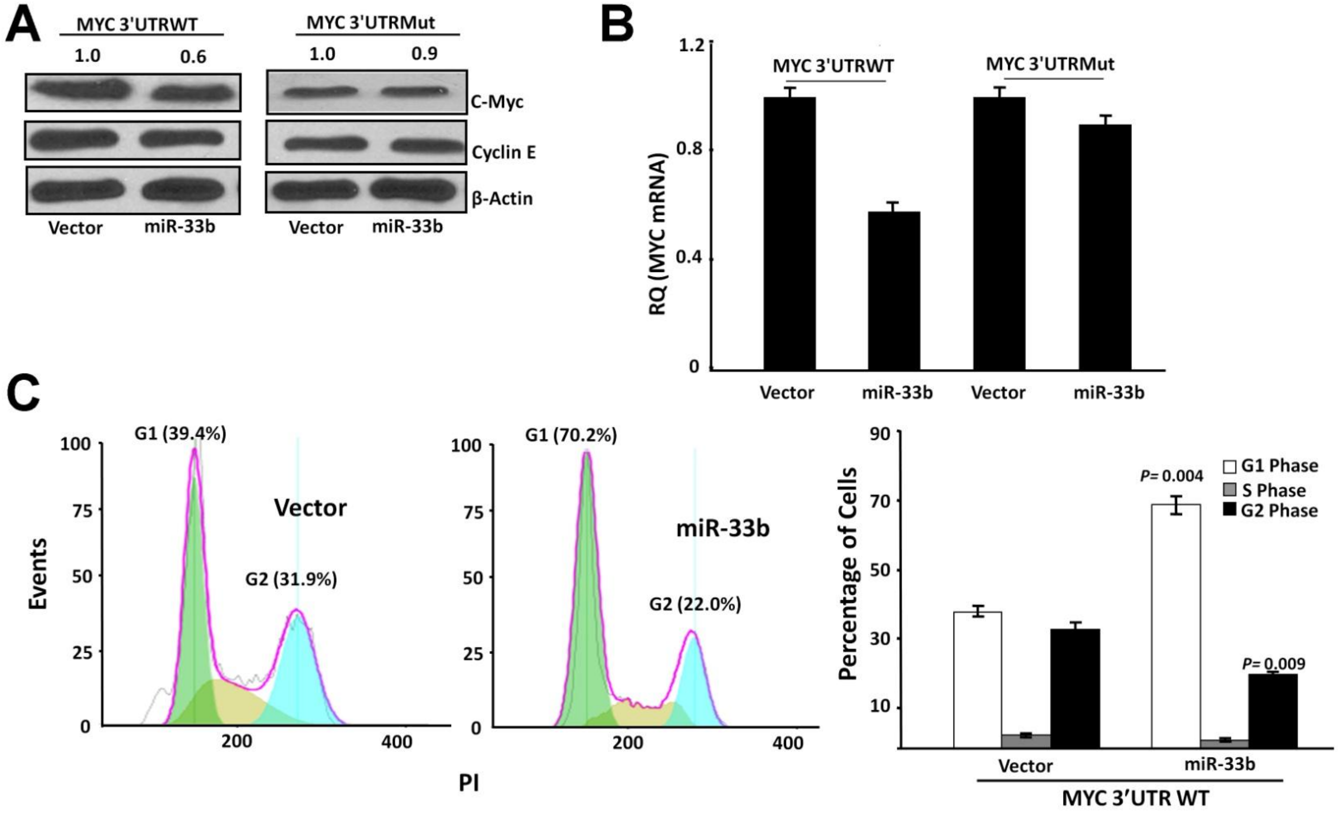

Figure S4. miR-33b down-regulates exogenously expressed c-Myc in *MYC*-null HO15.19 cells. (A) Western blotting analyses show that miR-33b down-regulates c-Myc exogenously expressed from a pcDNA-MYC construct with the 3'UTRWT and c-Myc transactivation target Cyclin E. miR-33b does not suppress exogenously expressed c-Myc with the 3'UTRMut lacking the miR-33b binding site. The numbers above the blot are the relative quantities of c-Myc normalized to β-actin. (B) qRT-PCR demonstrates a reduction in the MYC mRNA level following miR-33b overexpression. (C) Overexpression of miR-33b in HO15.19 cells with MYC and a WT 3'UTR increased G1 arrest. On the left are representative images of a single run with the y-axis denoting events (the number of cells) and the x-axis denoting the emitted fluorescence of the DNA dye (PI); a bar graph on the right is provided to summarize the three independent runs.

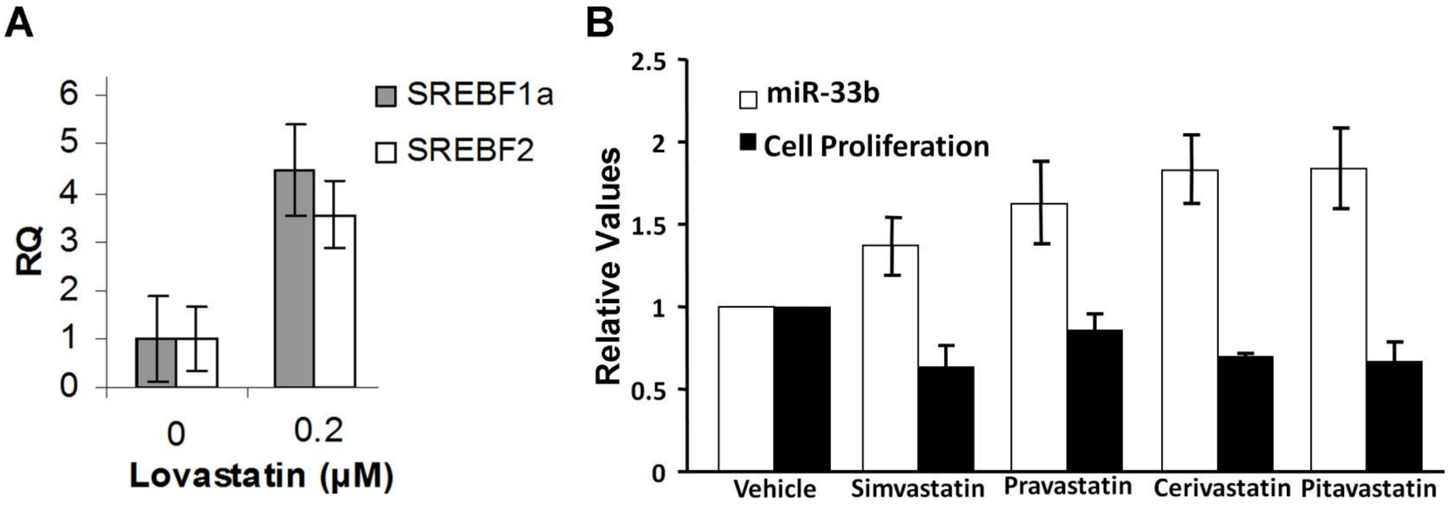

Figure S5. Statin affects gene expression in Daoy cells. (A) The qPCR analysis of SREBF1a and SREBF2 expression treated with 0.2 µM lovastatin. RQ, relative quantity normalized to β-actin. (B) Impact on cell proliferation and miR-33b expression by other statins. Daoy cells were treated with a 0.2 µM concentration of various statins (in DMSO) and cell proliferation and miR-33b levels were determined. Simvastatin, pravastatin, cerivastatin, and pitavastatin upregulated miR-33b expression. Relative values of miR-33b were normalized to U6 snRNA and those of cell proliferation were determined using the MTT assay.

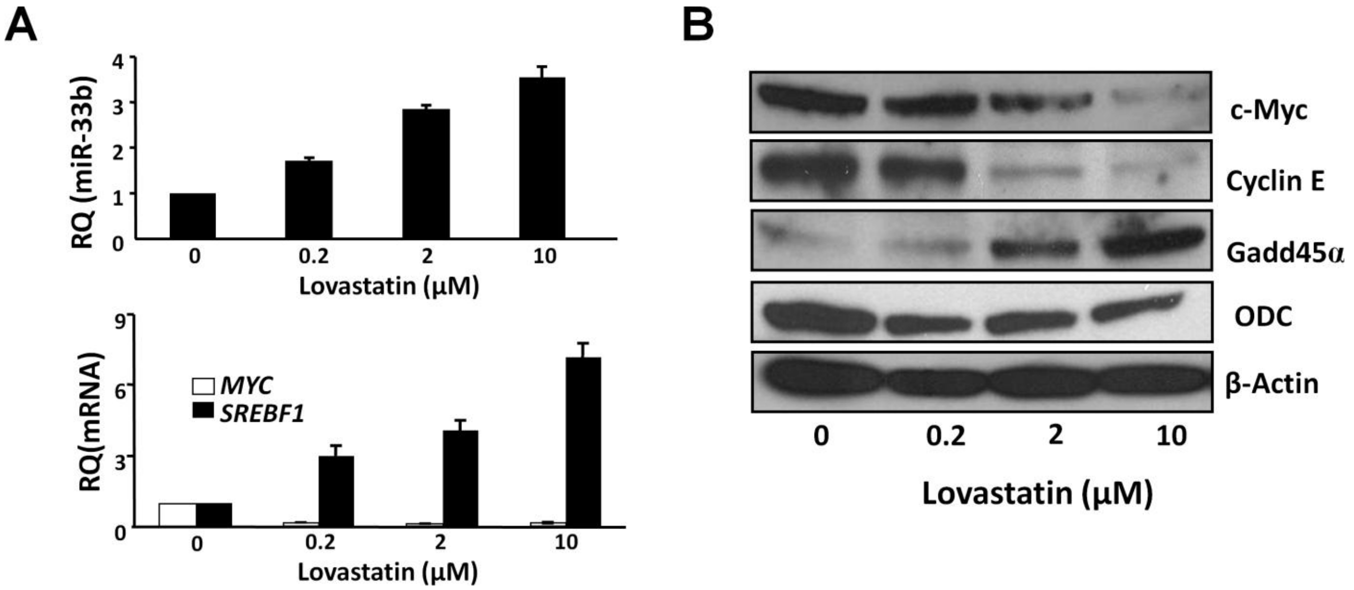

Figure S6. Lovastatin treatment for UW228 cells. (A) Lovastatin treatment increased the RNA levels of miR-33b and *SREBF1* and reduced *MYC* mRNA levels. (B) Western blotting analysis shows that increasing lovastatin concentration resulted in progressive down-regulation of c-Myc protein levels, accompanied by down-regulation of Cyclin E and ODC and upregulation of Gadd45α.

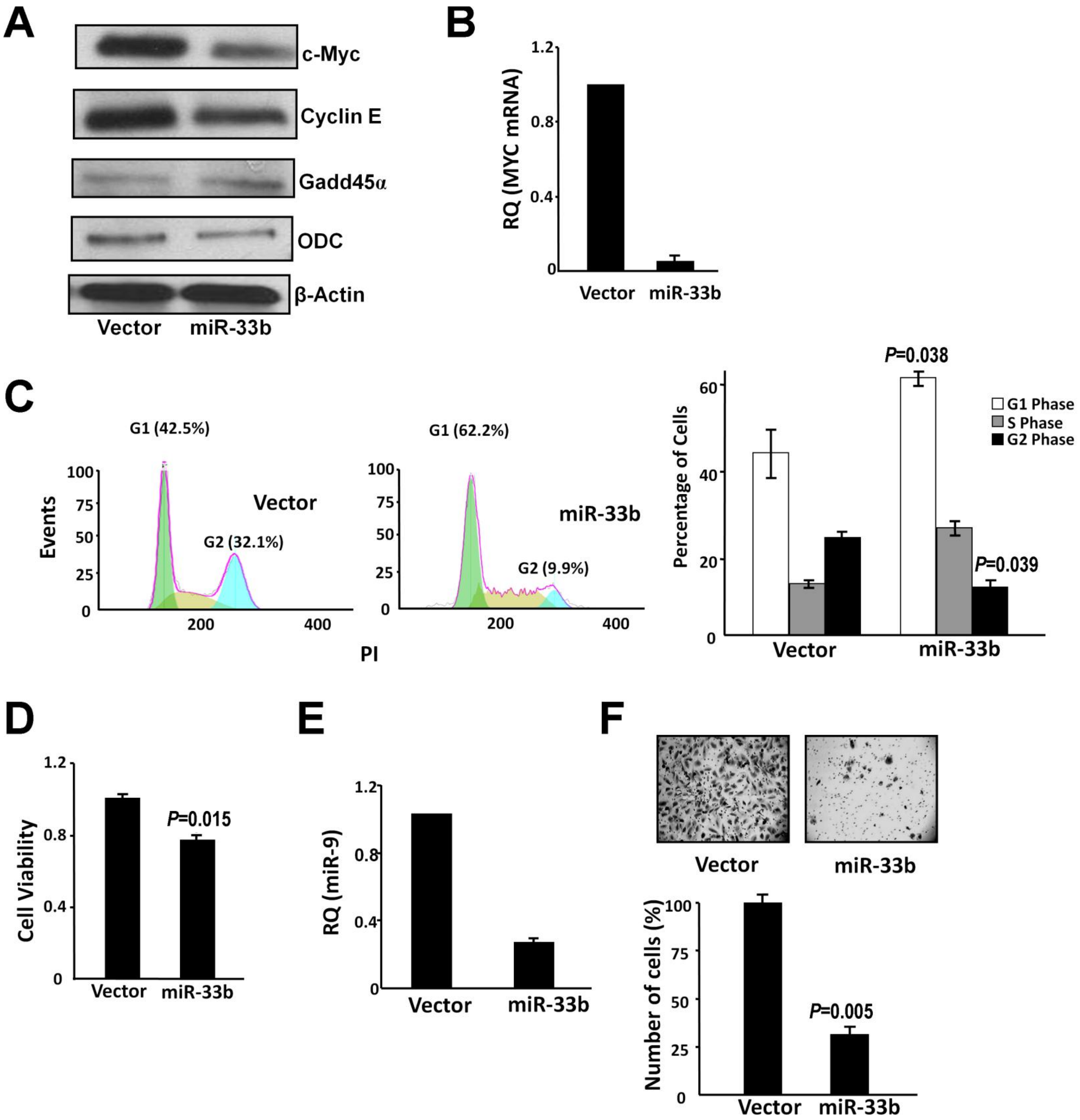

Figure S7. miR-33b overexpression in Daoy cells. (A) Protein levels of c-Myc and its transcriptional targets Cyclin E and ODC are down-regulated by miR-33b overexpression in Daoy cells. (B) miR-33b overexpression reduces *MYC* mRNA levels in Daoy cells. (C) miR-33b overexpression increases G1 cell cycle arrest in Daoy cells. On the left are representative images of a single run with the y-axis denoting events (the number of cells) and the x-axis denoting the emitted fluorescence of the DNA dye (PI); a bar graph on the right is provided to summarize the three independent runs. (D) miR-33b overexpression reduces cell proliferation. (E) The expression of miR-9 is down-regulated upon miR-33b overexpression. (F) The Transwell migration assay shows that miR-33b decreases cell migration of Daoy cells.

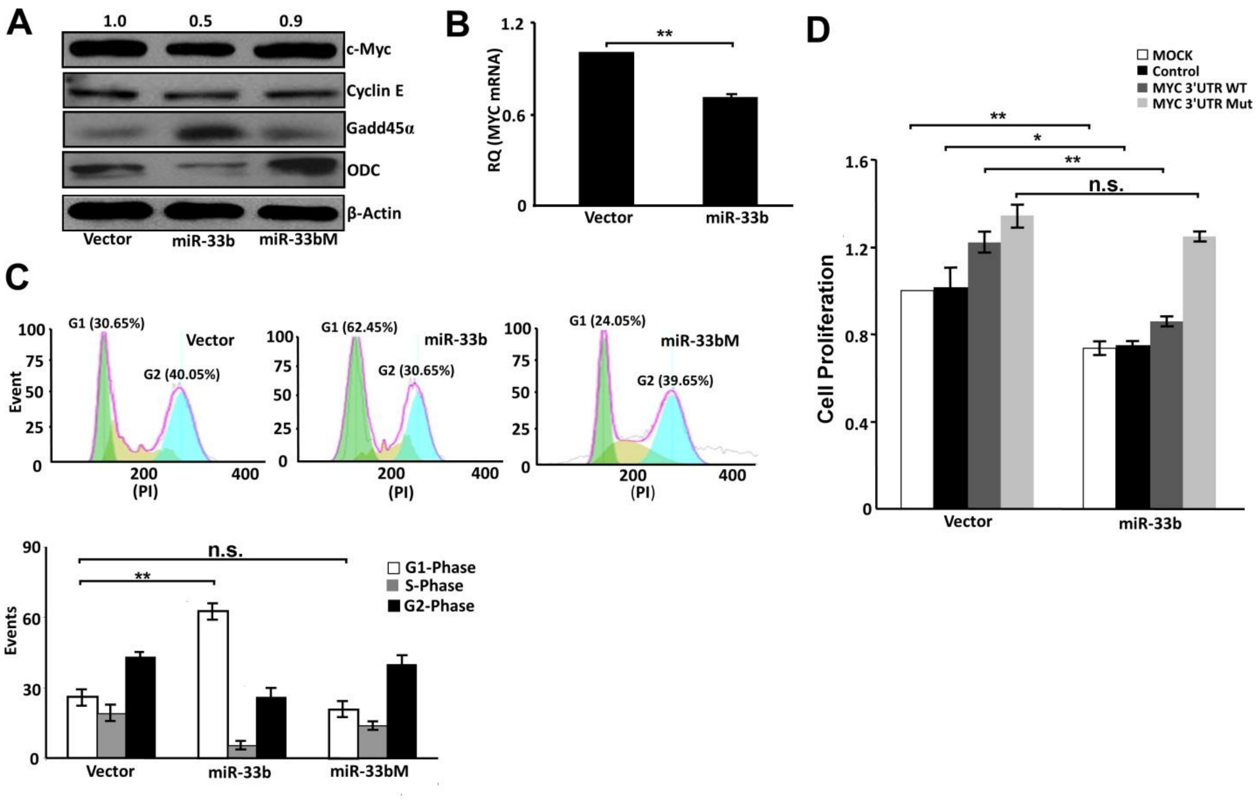

Figure S8. miR-33b down-regulates c-Myc in HeLa cells. (A) Immunoblotting analyses show that down-regulation of c-Myc and cyclin E and ODC and upregulation of Gadd45α by miR-33b. (B) Myc mRNA levels is reduced by miR-33b. (C) miR-33b, but not miR-33bM leads to increased G1 arrest. On the top are representative images of a single run with the y-axis denoting events (the number of cells) and the x-axis denoting the emitted fluorescence of the DNA dye (PI); a bar graph on the bottom is provided to summarize the three independent runs. (D) miR-33b decreases cell proliferation and this is likely due to c-Myc targeting as cell proliferation is not reduced when an exogenous c-Myc without miR-33b target sites in the 3'UTR is expressed. Cell proliferation is determined by the MTT assay. MOCK: cells are only transfected with miR-33b or its parental vector; Control: cells are co-transfected with an empty expression plasmid (pCDNA3; Invitrogen). *, $P \leq 0.05$; **, $P \leq 0.01$; n.s., not significant.

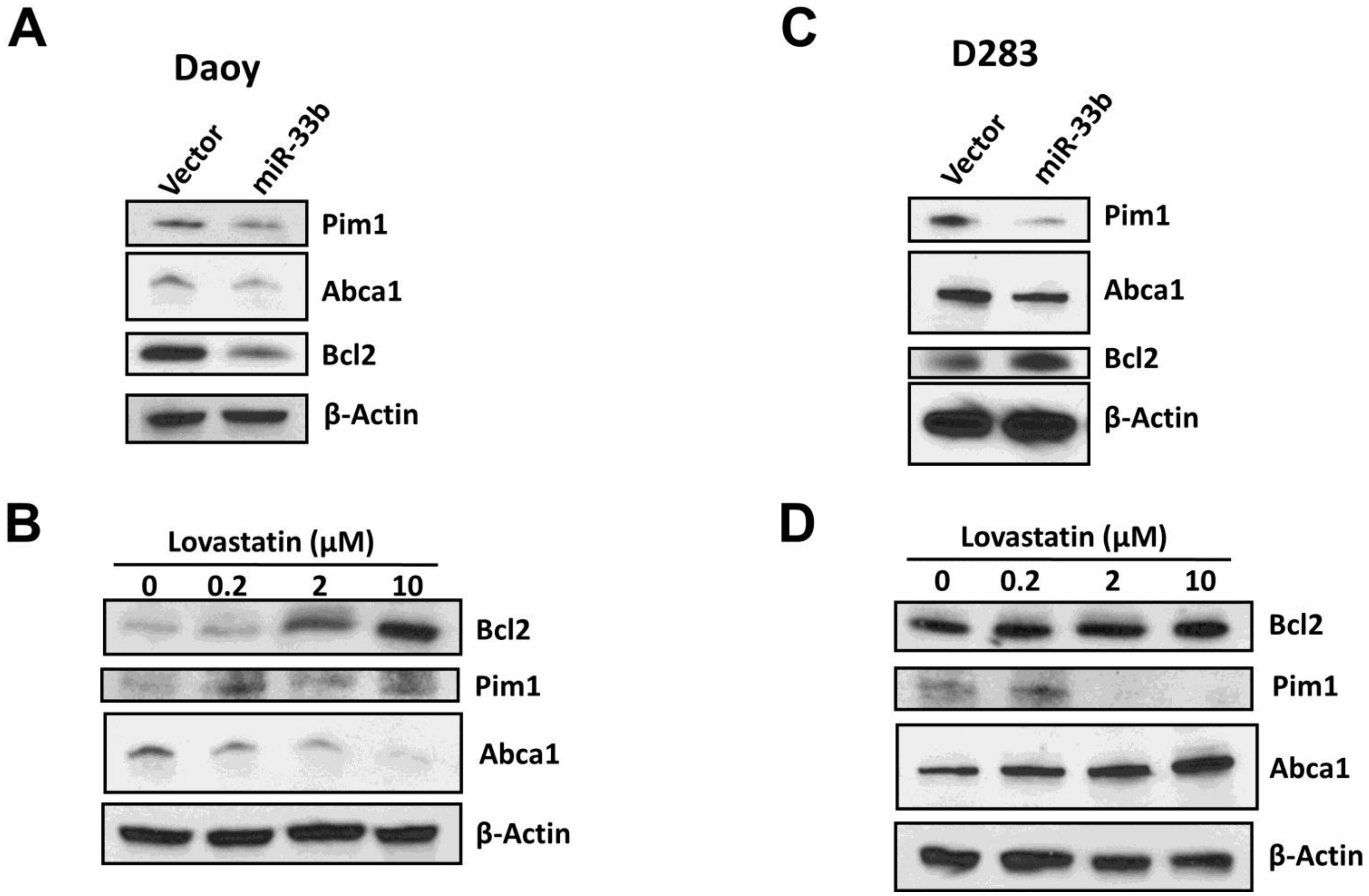

Figure S9. miR-33b regulates the expression of Abca1 and Pim1 in medulloblastoma cells. (A) (C), overexpression of miR-33b. (B) (D), cells treated with lovastatin. (A) (B), Daoy cells. (C) (D), D283 cells.

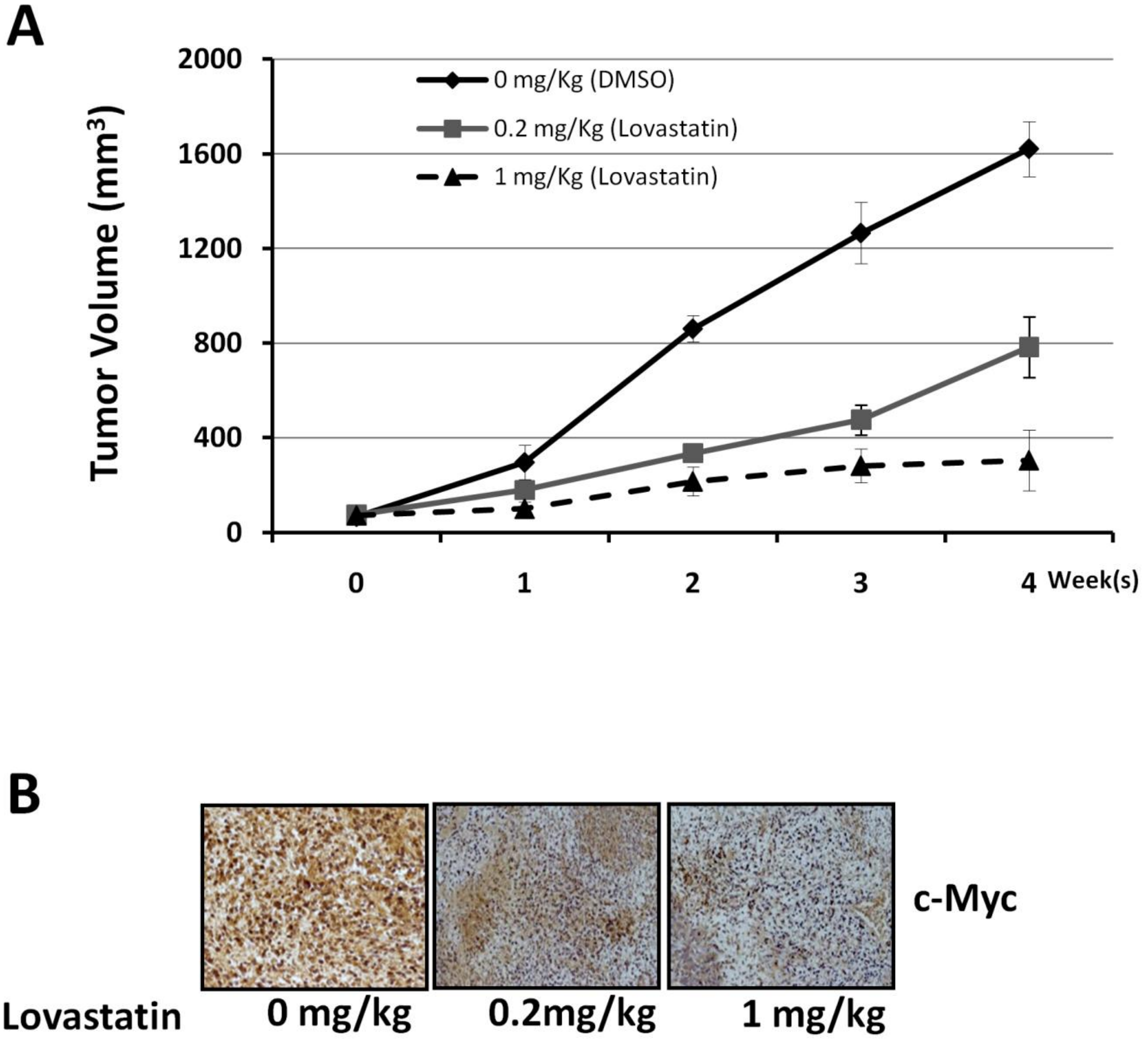

Figure S10. Subcutaneous xenograft assay of Daoy cells treated with lovastatin. (A) Volumes of xenograft tumors. (B) IHC analyses of c-Myc expression in tumor sections.

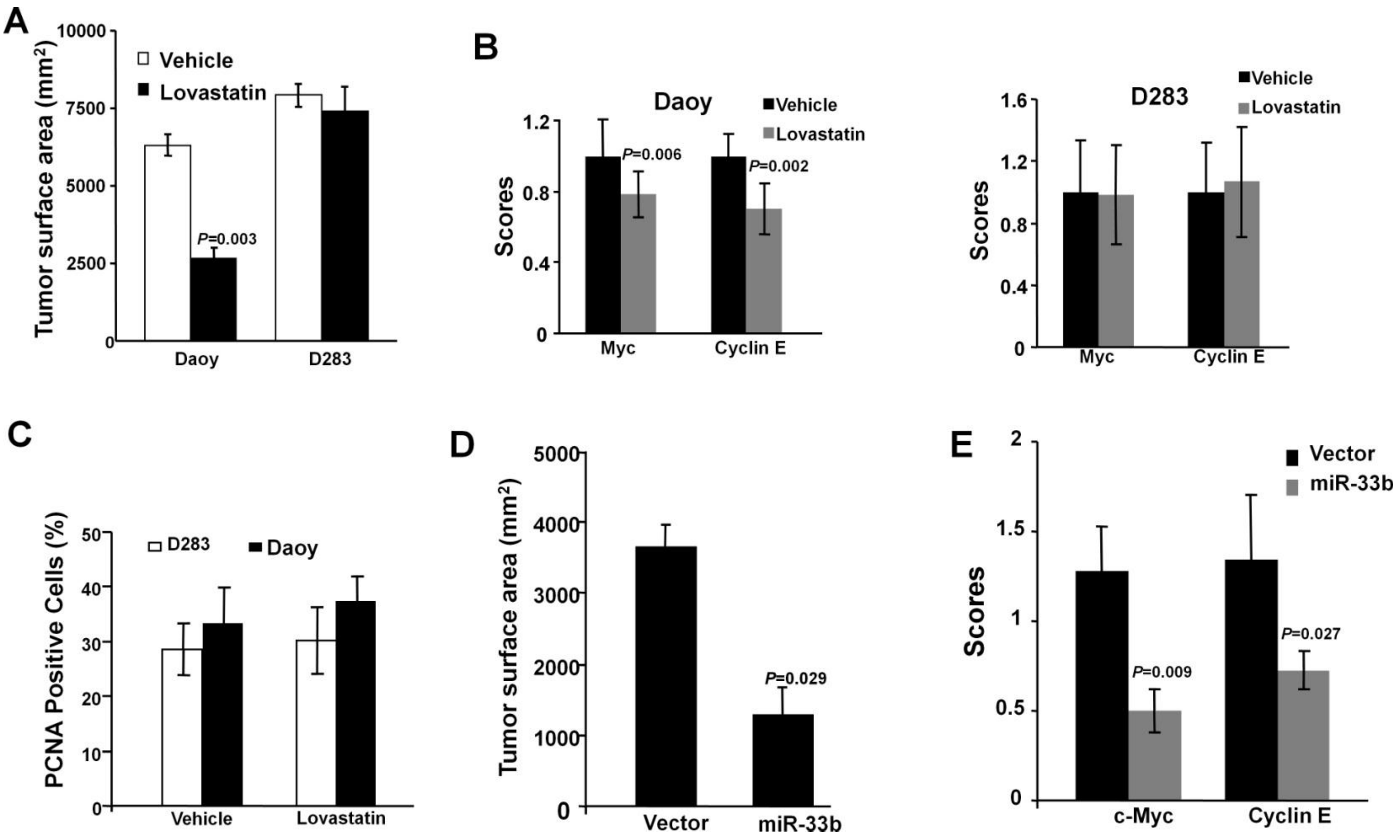

Figure S11. Pathological analyses of orthotopic xenograft tumors in mice. (A) Lovastatin treatment reduces the maximum surface area of tumors xenografted with Daoy cells but not that with D283 cells. (B) Lovastatin treatment reduces the expression of c-Myc and Cyclin E in tumors xenografted with Daoy cells but not that with D283 cells. (C) Lovastatin treatment does not affect the percentage of PCNA-positive cells in tumors xenografted with Daoy cells or that with D283 cells. (E) Reduced maximum surface area of tumors xenografted with D283 cells overexpressing miR-33b, compared with that of cells carrying the parental vector.

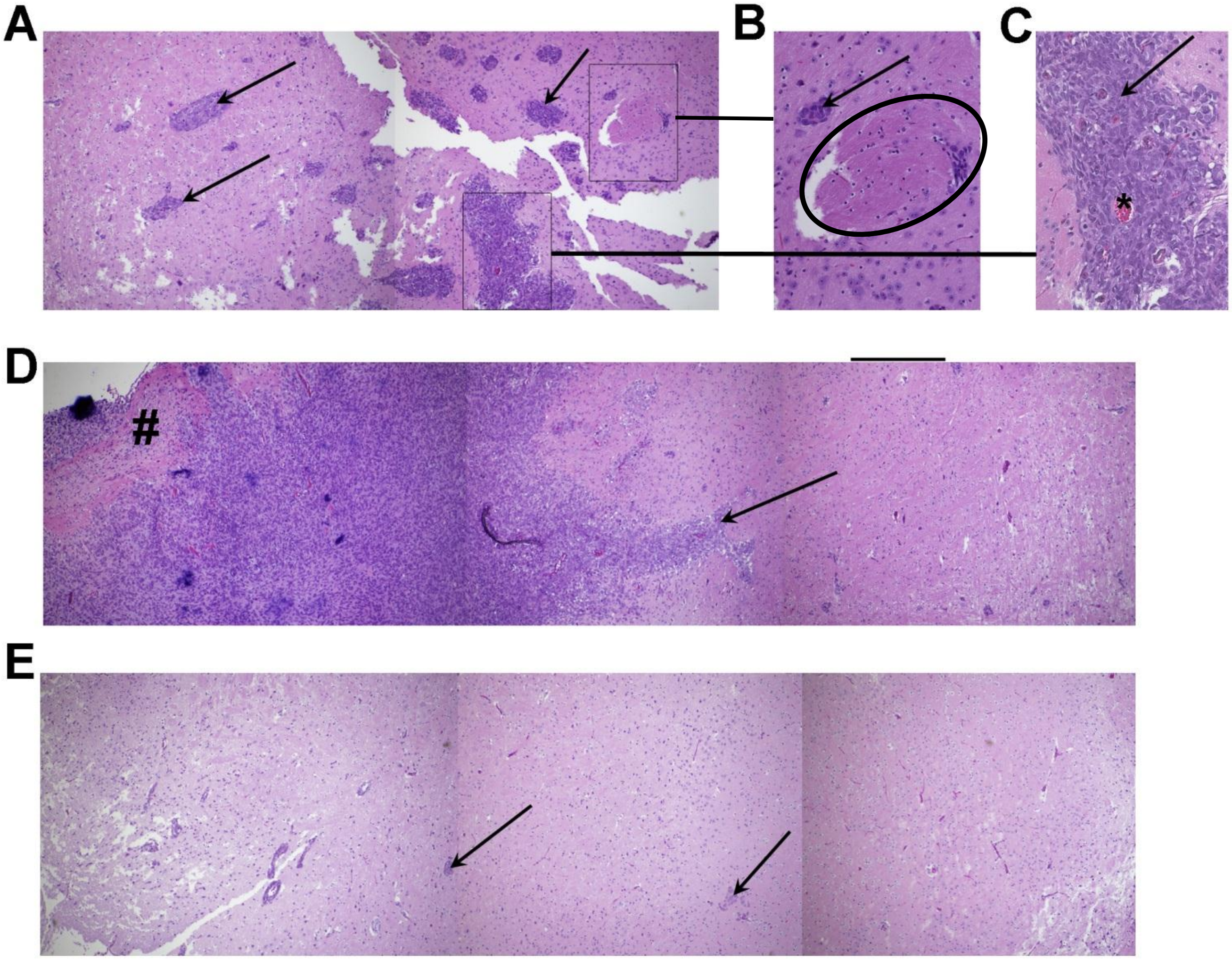

Figure S12. Histology of mice xenografted with Daoy and treated with lovastatin. Brain sections were stained with H&E, and representative images were compared. Arrows point to tumor cells. (A-D) H&E staining of an animal treated with vehicle. “*” indicates newly-formed blood vesicle. “#” indicates misplaced white matter. Swollen neurons are circled in (B). (E) Tumor cell invasion into the cerebral cortex in mice treated with lovastatin. Brain sections were from two mice that died around the same time (day 52 from the control group, Panel A-D, and day 53 from the treated group, Panel E).

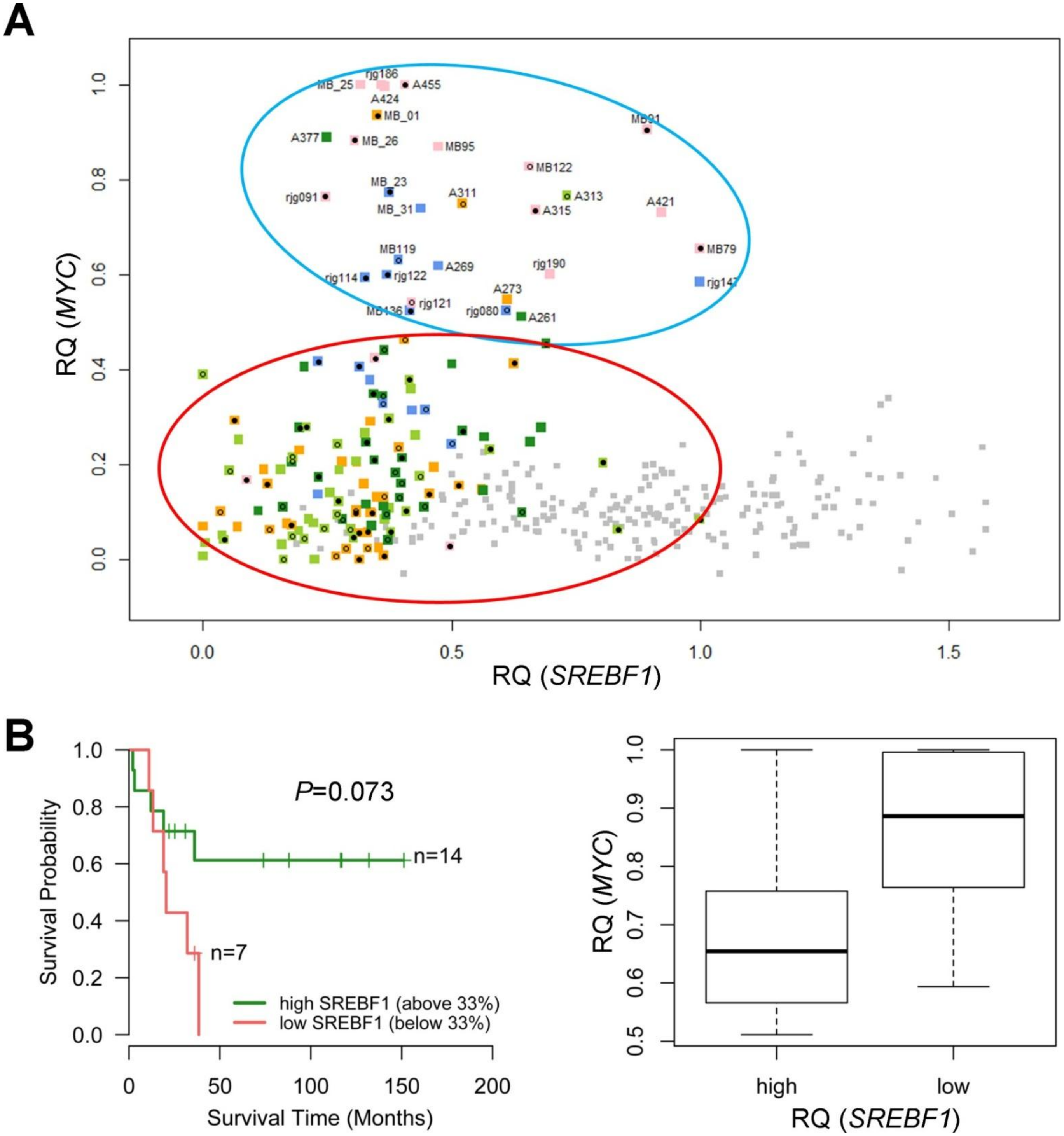

Figure S13. Negative correlation of *MYC* and *SREBF1* expression in medulloblastoma. (A) The biplot of relative levels of the mean expression of *MYC* and *SREBF1*. Gray color indicates normal brain and other colors indicate tumors. (B) A Kaplan–Meier curve and the log-rank test for overall survival of medulloblastoma patients (21 out of these 29 cases have survival data). A box-plot on the right shows the negative correlation of Myc and Srebf1 expression in 21 patients. RQ, relative quantitation.

Supplemental Table S1 FDA-approved drugs that upregulate miR-33b in Daoy cells

Compounds	miR-33 upregulation (fold)	FDA approved for cancer
Lovasatin	2.2	No
Carisoprodol	2.2	No
Albendazole	2.0	No
Memantine	2.7	No
Lincomycin	2.0	No
5-Azacytidine	3.3	Yes
Celecoxib	2.4	Yes
Docetaxel	2.3	Yes
Epirubicin	2.2	Yes
Triptolide	4.3	Yes
Dactinomycin	3.9	Yes
Vincristine	2.5	Yes
